# “Membrane‐Guided” Repair Strategy: Precision Delivery of GGT1 Degrader for Targeted Repair and Regeneration of Spinal Cord Neurons

**DOI:** 10.1002/advs.75554

**Published:** 2026-05-14

**Authors:** Tao Yang, Lei Ye, Peigen Xie, Tao Song, Jian Chen, Heng Zhao, Zhengshan Liu, Xi Chen, Jianing Ding, Xintian Ding, Ao Shao, Miaomiao Wu, Fengdong Zhao, Siyue Tao, Tao You

**Affiliations:** ^1^ Department of Orthopedics The First Affiliated Hospital of USTC Division of Life Sciences and Medicine University of Science and Technology of China Hefei China; ^2^ Department of Orthopedic Surgery Sir Run Run Shaw Hospital Zhejiang University School of Medicine Hangzhou Zhejiang China; ^3^ Department of Orthopedic Surgery Shanghai Sixth People's Hospital Affiliated to Shanghai Jiao Tong University School of Medicine Shanghai China; ^4^ Department of Spine Surgery The Third Affiliated Hospital of Sun Yat‐Sen University Macau Medical Science & Technology Research Association Guangzhou China; ^5^ Department of Psychiatry University of Texas Southwestern Medical Center Dallas Texas USA; ^6^ Department of Orthopedics The Affiliated Chuzhou of Anhui Medical University Chuzhou China

**Keywords:** enocyanin, ferroptosis, GGT1, membrane delivery system, spinal cord injury

## Abstract

Ferroptosis is one of the important mechanisms of secondary neuronal death after spinal cord injury (SCI). However, the upstream regulators that could be targeted for therapeutic intervention remain poorly defined. This study identifies gamma‐glutamyl transferase 1 (GGT1) as a key driver of ferroptosis, upregulated in neurons post‐SCI. Screening a 150‐compound natural product library, we discovered Enocyanin (EA), which reduced GGT1 protein levels, protected neurons from hypoxic injury, and exhibited anti‐ferroptotic effects. Mechanistically, EA promoted GGT1 degradation through the E3 ligase MGRN1, leading to K48‐linked polyubiquitination and proteasomal clearance, halting ferroptosis. To improve EA's stability and delivery, we engineered a biomimetic nanoplatform (NSCm@EA) using neural stem cell membranes, enhancing drug accumulation at the injured spinal cord. At single‐cell resolution, NSCm@EA was shown to precisely remodel neuronal subpopulations, selectively expanding γ‐motor neurons and upregulating synaptic genes such as Gria2 and Negr1, while suppressing inflammatory and oxidative stress pathways. In summary, this study reveals GGT1's role in ferroptosis, identifies a natural product that induces its ubiquitin‐mediated degradation, and presents a targeted biomimetic delivery strategy for precise intervention in spinal cord injury.

## Graphical abstract

This study confirms that GGT1 is a key driver of neuronal ferroptosis following spinal cord injury. We developed NSCm@EA, a biomimetic delivery system coated with neural stem cell membranes, for precise delivery of enocyanin to injured neurons. By combining targeted delivery with ubiquitination degradation mechanisms, this system promotes MGRN1‐mediated ubiquitination and proteasomal degradation of GGT1 K48 junctions, thereby inhibiting ferroptosis, remodeling neuronal subpopulations, and facilitating neural functional recovery.

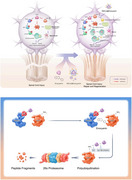

## 1 Introduction

Spinal cord injury (SCI) results in severe neurological dysfunction due to progressive neuronal loss from the initial trauma and a subsequent disruption of the spinal cord microenvironment [1]. While emergency surgery can alleviate mechanical compression, the persisting pathological environment severely limits the effectiveness of pharmacological interventions, posing a significant challenge to functional recovery [2].

Although high‐dose methylprednisolone was once considered the standard treatment, its severe systemic side effects preclude routine use [3, 4, 5]. Since the primary mechanical injury is often irreversible, contemporary therapeutic strategies now focus on protecting the surviving neurons and promoting their regeneration [6, 7, 8]. Extensive research has shown that classical cell death pathways, such as apoptosis and necrosis, play a role in SCI [9, 10, 11]. More recently, ferroptosis—an iron‐dependent, lipid peroxidation‐driven form of regulated cell death—has emerged as a key driver of post‐injury neuronal loss, offering a promising new target for neuroprotection [12, 13, 14]. However, a major challenge remains: identifying the specific molecular regulators of ferroptosis in spinal neurons and developing targeted interventions.

Gamma‐glutamyl transferase 1 (GGT1), a key enzyme in glutathione (GSH) metabolism, is central to cellular redox homeostasis [15, 16, 17]. Our preliminary work revealed a time‐dependent upregulation of GGT1 in neurons after SCI. Contrary to the assumed protective role of GGT1, elevated activity of this enzyme depletes GSH, exacerbates oxidative stress, and actively promotes neuronal ferroptosis. This positions GGT1 as a critical molecular link between injury stress and ferroptotic cell death, making it an attractive therapeutic target. Yet, the lack of effective agents to inhibit GGT1 has hindered progress in this area.

To address this gap, we conducted a systematic pharmacological screen of a large natural product library. From this, we identified Enocyanin (EA), a natural anthocyanin derived from grape skin, as a promising candidate. While anthocyanins are known for their anti‐inflammatory and antioxidant properties, their role in SCI has not been well explored. Our results demonstrate that EA significantly reduces neuronal damage in vitro and improves functional recovery in a murine SCI model. Mechanistically, EA does not suppress GGT1 transcription but uniquely promotes the ubiquitination and subsequent proteasomal degradation of the GGT1 protein. This mechanism provides a novel, source‐level suppression of GGT1‐mediated ferroptosis, establishing a new approach for targeting protein stability in neuroprotectants.

However, effective therapeutic delivery to the injured spinal cord remains a major challenge [18, 19]. The pathological microenvironment at the injury site—characterized by a disrupted blood‐spinal cord barrier (BSCB), inflammatory infiltration, and intense oxidative stress—severely hampers drug delivery and retention [20]. This hostile environment also undermines other advanced therapeutic strategies. For instance, stem cell transplantation remains largely confined to preclinical studies due to low cell survival and differentiation rates [21, 22, 23]. Similarly, gene therapies face challenges from potential immune recognition and neutralization, limiting their clinical translation [24]. Regulating microenvironment homeostasis is crucial for SCI repair [25]. While EA itself exhibits low toxicity, its instability and poor bioavailability further compromise its in vivo efficacy [26].

Inspired by the injury‐responsive and immunomodulatory properties of neural stem cells (NSCs) [27, 28, 29], we developed a biomimetic delivery platform to overcome these barriers. We constructed NSC membrane‐camouflaged nanovesicles encapsulating EA (NSCm@EA). This nanostructure facilitates preferential accumulation at the injury site, potentially through interactions mediated by adhesion molecules on the NSC membrane, which could enhance local delivery efficiency.

In summary, this work delineates the central role of GGT1 in driving post‐SCI ferroptosis and its upstream regulatory mechanism via ubiquitination. We further identify EA as the first natural product capable of inducing GGT1 degradation via the proteasome. Finally, we engineer a biomimetic targeted delivery system to solve the critical challenge of efficient drug transport to the injured cord, aiming to provide new ideas for developing promising treatment strategies for SCI.

## 2 Results

### 2.1 Temporal Dynamics of GGT1 Upregulation Parallel Enhanced Ferroptosis After SCI

**FIGURE 1:**
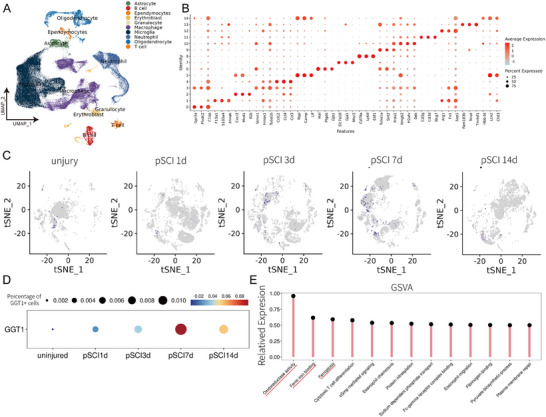
Elevated expression of GGT1 correlates with increased ferroptosis levels following spinal cord injury. (A) UMAP visualization of spinal cord cell landscapes at different time points post‐injury, identifying 10 cell populations. Colors represent distinct cell clusters. (B) Dot plot of DEGs for each cluster, with dot color indicating the average RNA expression. (C) tSNE showing GGT1 expression across various cell types at different time points after injury. (D) Dot size represents the proportion of GGT1‐positive cells relative to the total cell count. (E) Bar graph showing upregulated GSVA entries in pSCI1d compared to the uninjured group.

To investigate the intrinsic factors underlying neuronal death following spinal cord injury (SCI), we conducted an in‐depth analysis of Single‐cell sequencing data from Sun et al. [30]. to explore the pathophysiological changes in neurons at various time points post‐SCI (Figure 1A,B). The analysis showed a rapid increase in GGT1 expression within 1 days after spinal cord injury, followed by a time‐dependent rise over the first 7 days, after which it declined (Figure 1C,D). Subsequently, we performed pathway enrichment analysis on differentially expressed genes from the temporal SCI data. GSVA enrichment analysis of differentially expressed genes revealed higher levels of oxidoreductase activity and ferroptosis in the injured spinal cord compared to the undamaged tissue (Figure 1E).

**FIGURE 2:**
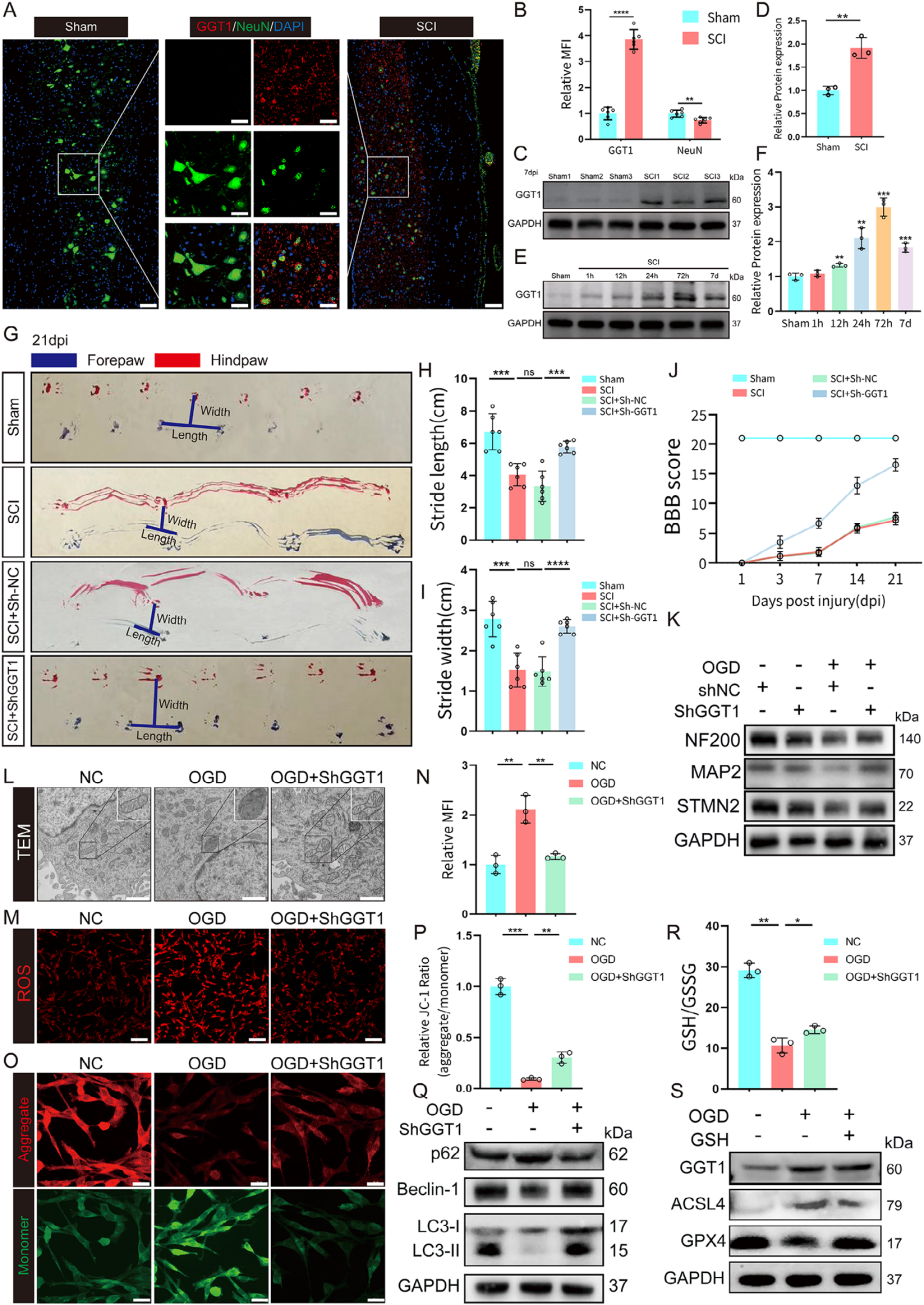
GGT1 knockdown promotes functional recovery in vivo and attenuates ferroptosis in vitro. (A) Representative IF images of GGT1 (red) and NeuN (green) expression in sham and SCI groups. Scale bars: 100 or 50 µm (B) Quantitative analysis of GGT1 and NeuN fluorescence intensity (n = 6). (C, D) WB detection of GGT1 expression in spinal cord lysates from sham and SCI models 7 days post‐SCI. (E,F) WB analysis of GGT1 expression in spinal cord lysates at specific time points after sham and SCI modeling. (G) Analysis of gait with representative footprints at 21 days after SCI. (H,I) Statistical Analysis of Stride Length and Stride Width (n = 6). (J) BBB scores at 1,3,7,14, and 21 days after SCI (n = 6). (K) WB detection of GGT1 knockdown in OGD models, along with expression of neuronal and axonal marker proteins (NF200, MAP2, STMN2). (L) The morphology of mitochondrial cristae in PC12 cells was observed by transmission electron microscopy after knockdown of GGT1 in the OGD model. (M,N) Determination of ROS, Scale bar: 100 µm. (O, P) Mitochondrial membrane potential was determined by the JC‐1 method. Scale bar:20 µm. (Q) WB detected autophagy marker protein expression level. (R) After OGD modeling, the rati` o of GSH/GSSG was quantitatively analyzed following either EA treatment or no EA treatment. (S) In vitro supplementation of GSH after OGD modeling in WB assay for the expression of GGT1 and ferroptosis‐related proteins ACSL4 and GPX4. Data are expressed as mean ± SD, All are comparisons between each group and control, *: *p* < 0.05, **: *p* < 0.01, and ***: *p* < 0.001.

Prompted by this transcriptional signature, we assessed GGT1 expression in vivo at 7 days post‐SCI. Immunofluorescence analysis confirmed a decrease in the proportion of NeuN‐positive neurons within the lesioned spinal cord, paralleled by a striking 3.12‐fold upregulation of GGT1 protein in these cells (Figure 2A,B). Consistent with this, western blot analysis of spinal cord tissue lysates at the same time point showed a corresponding increase in GGT1 protein levels (Figure 2C,D). Notably, this upregulation occurred early, with GGT1 protein levels beginning to rise as early as 12 h after injury and nearly doubling by 72 h (Figure 2E,F).

Collectively, these data demonstrate that injured spinal cord neurons exhibit enhanced ferroptosis alongside a marked and progressive increase in GGT1 expression, suggesting a potential mechanistic link between the two phenomena.

### 2.2 GGT1 Upregulation Acts as a Key Driver of Neuronal Ferroptosis in SCI

Building on these findings, we hypothesized a positive correlation between elevated GGT1 expression in injured neurons and the extent of ferroptosis following SCI. To investigate the functional role of neuronal GGT1 post‐injury, we used a lentiviral vector to delivery shGGT1 to knock down its expression in mice.

Behavioral analysis after SCI induction revealed significantly shortened stride length and width, along with a Basso, Beattie, Bresnahan (BBB) score of zero on 1 day post‐injury (dpi), consistent with successful and stable model establishment. In contrast, GGT1 knockdown markedly improved locomotor recovery (Figure 2G–I). Mice in the shGGT1 group exhibited not only greater stride length and width compared to the shNC controls but also a substantially higher BBB score, reaching 16.5 by day 21 (Figure 2J). These results suggest that GGT1 depletion effectively restores hindlimb motor function and coordination after SCI.

Since ischemia and hypoxia are hallmark pathophysiological features of SCI, we modeled neuronal injury in vitro using oxygen‐glucose deprivation (OGD). OGD treatment markedly suppressed the expression of key neuronal proteins including neurofilament 200 (NF200), microtubule‐associated protein 2 (MAP2), and stathmin 2 (STMN2), an effect that was partially reversed upon GGT1 silencing (Figure 2K). Furthermore, GGT1 knockdown in PC12 cells improved mitochondrial morphology (Figure 2L), reduced mitochondrial membrane potential (Figure 2O,P), and consequently lowered intracellular reactive oxygen species (ROS) levels (Figure 2M,N). We also observed that GGT1 silencing was associated with increased LC3‐II conversion and reduced p62 levels (Figure 2Q). As anticipated, OGD treatment dramatically decreased reduced glutathione (GSH) levels due to intensified oxidative stress. Notably, upregulated GGT1 expression further hydrolyzes GSH. Consequently, its knockdown increased the GSH/GSSG ratio by 46% compared to the OGD group (Figure 2R). In subsequent rescue experiments, although exogenous GSH supplementation did not inhibit GGT1 expression, it effectively promoted levels of the ferroptosis‐inhibiting protein GPX4 while suppressing expression of the lipid transporter ACSL4 (Figure 2S).

Taken together, these results indicated that the neurons suffered from ferroptosis after SCI, and GGT1 depletion effectively restores promotes functional recovery and attenuates ferroptosis.

### 2.3 Identification of EA as a Neuroprotective Agent from a Transformation‐Oriented Screening

**FIGURE 3:**
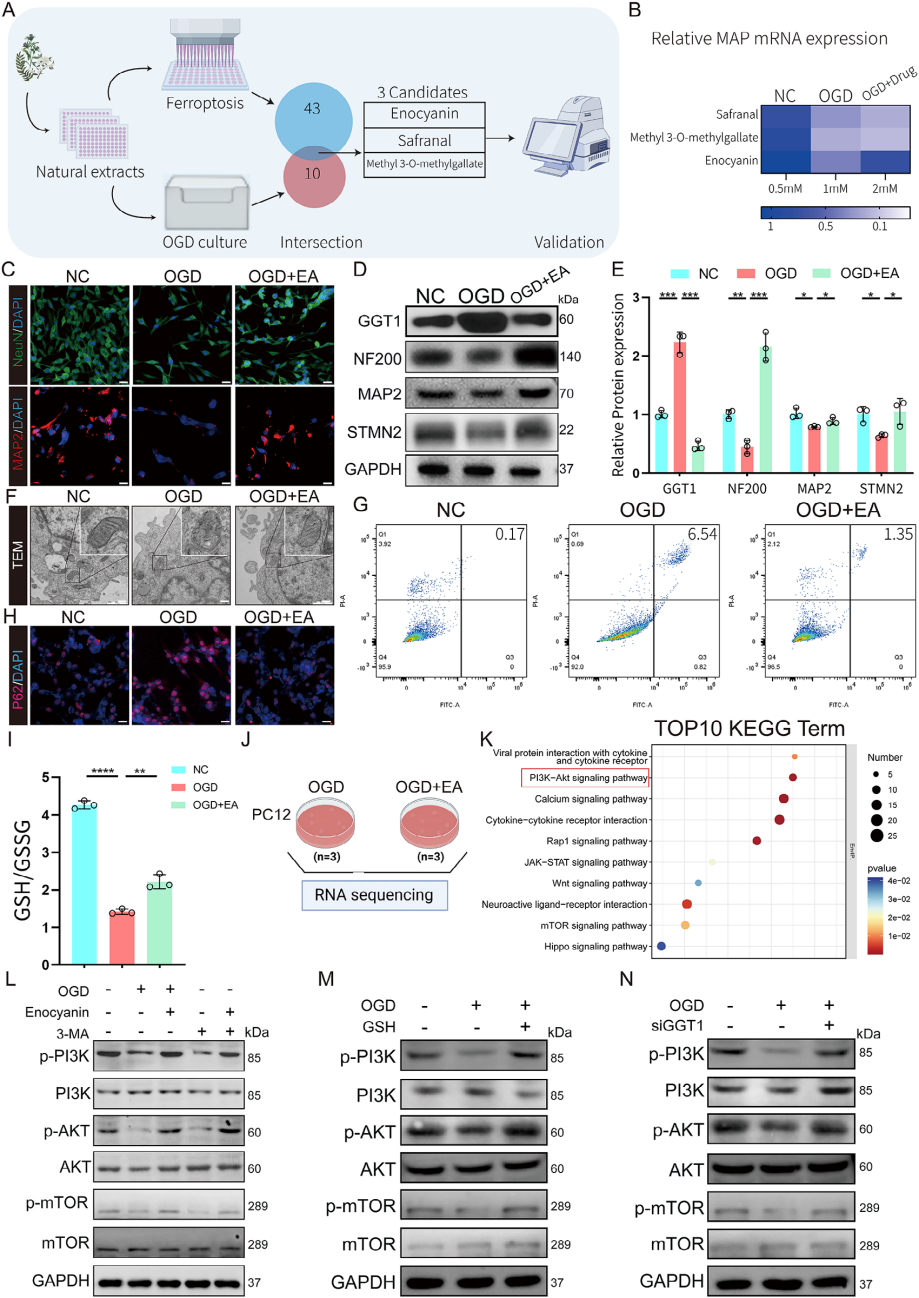
EA attenuates ferroptosis in PC12 cells induced by OGD via the PI3K‐AKT signaling pathway. (A) Schematic diagram of pioneer compound screening. (B) Heatmap display of candidate compounds regulating MAP2 mRNA. (C) IF staining tested Neun (green‐Coralite488), MAP2(red‐CoraLite594) in EA processing of PC12 cells. Scale bars: 20 µm. (D) WB detected PC12 cells regeneration of axons markers MAP2, STMN2, NF200 expression level of proteins. (E) Quantitative analysis of the ratio of GGT1, MAP2, STMN2, and NF200 (n = 3). (F) TEM method determination of mitochondria, Scale bars:2 or 1 µm. (G) Flow cytometry analysis of PC12 cell apoptosis. Apoptosis was analyzed by annexin V staining and PI. (H) IF staining tested p62 (red‐CoraLite594) in EA processing of PC12 cells. Scale bars: 20 µm. (I) Statistical analysis of the GSH/GSSG ratio in PC12 cells under OGD culture with or without EA. (J) Schematic diagram of pre‐cell processing for RNA‐seq. (K) KEGG pathway enrichment analysis of RNA‐seq results from PC12 cells after OGD modeling, with or without EA treatment. (L–N) WB detected p‐PI3K, and AKT, p‐AKT, mTOR, p‐mTOR protein expression levels. Data are expressed as mean ± SD, All are comparisons between each group and control, *: *p* < 0.05, **: *p* < 0.01, and ***: *p* < 0.001.

To facilitate translation, we conducted a drug screening using a library of 150 natural product compounds. From an initial set of 43 candidates with anti‐ferroptotic activity and 10 showing neuroprotective effects against OGD, only 3 compounds emerged from the intersection (Figure 3A; Figure S1A,B). Among these, EA uniquely enhanced the expression of axonal regeneration markers, including MAP2 and STMN2, in PC12 cells (Figure 3B; Figure S1C).

Since EA is a newly characterized anthocyanin (Figure S2A;), we first assessed its biosafety, observing no cytotoxicity in PC12 cells at concentrations up to 0.5 mg/mL using CCK‐8 assays (Figure S2B). In the OGD model, EA significantly increased the fluorescence intensity of neuronal and axonal markers (NeuN, MAP2, STMN2), restored the expression of regeneration‐related proteins suppressed by OGD, and—as anticipated—effectively inhibited the injury—induced upregulation of GGT1 protein (Figure 3C–E; Figure S5A). Transmission electron microscopy further revealed that EA mitigated OGD‐induced damage to mitochondrial cristae (Figure 3F). Consistent with these morphological improvements, EA suppressed the expression of pro‐inflammatory and pro‐apoptotic mRNA, resulting in a 67.1% reduction in p62 fluorescence intensity, further demonstrating its anti‐apoptotic effect. Additionally, the proportion of dead neurons decreased to 20.2% compared to the OGD group (Figure 3G,H; Figure S5B). As a potential GGT1 inhibitor, we found that EA supplementation in vitro increased the GSH/GSSG ratio (Figure 3I).

Next, we validated the therapeutic effect of EA in vivo using a murine SCI model (Figure S3A). EA treatment markedly improved hindlimb motor function, as evidenced by a BBB score of 9.83 and a climbing angle of 26.3° at 21 dpi (Figure S3B,C). Gait analysis showed that stride length and width recovered to 72.1% and 65.9% of sham levels, respectively (Figure S3D–F). Histological analysis of spinal cord sections revealed that EA reduced the lesion area by 27.6%, preserved Nissl bodies in neurons (Figure S3G–I), and increased the expression of axonal regeneration marker proteins, consistent with the in vitro results (Figure S3J,K).

While these in vitro and in vivo results establish EA's role in promoting axonal regeneration and inhibiting neuronal apoptosis, the precise mechanism by which EA modulates neuronal function through GGT1 remains unclear. To investigate this, we performed a transcriptomic analysis (Figure 3J; Figure S4). KEGG enrichment analysis pointed to a significant influence of EA on the PI3K/AKT signaling pathway (Figure 3K). Given our prior observation that GGT1 knockdown promotes neuronal autophagy, we hypothesized that EA, by inhibiting GGT1, activates the PI3K/AKT/mTOR pathway in injured neurons. Notably, this activation was accompanied by changes in autophagy‐related proteins (LC3‐II increase, p62 decrease), suggesting a complex interplay between mTOR signaling and autophagic responses that may involve non‐canonical pathways or compensatory mechanisms. Supporting this hypothesis, EA retained its ability to activate this pathway even after PI3K inhibition by 3‐MA (Figure 3L; Figure S5C). Furthermore, supplementing with GSH, the substrate of GGT1 (Figure 3M; Figure S5D), in vitro recapitulated the pathway activation observed with GGT1 knockdown (Figure 3N; Figure S5E), reinforcing the link between GGT1 suppression and the pro‐regenerative PI3K/AKT/mTOR/autophagy axis.

### 2.4 MGRN1 Mediates the Ubiquitination and Degradation of GGT1

**FIGURE 4:**
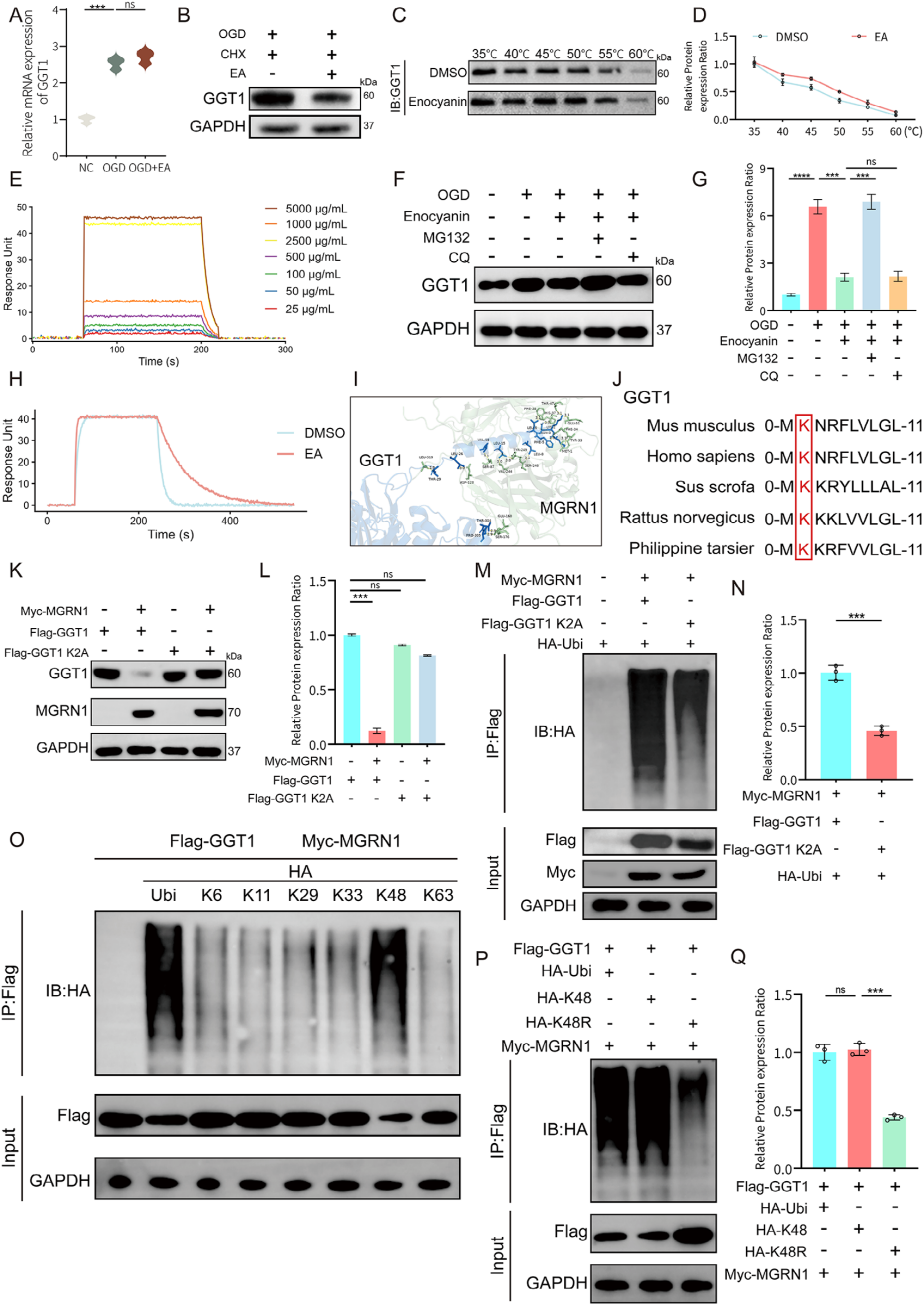
MGRN1 promotes GGT1 ubiquitination by binding to the GGT1 K2 ubiquitination site via the K48 ubiquitin chain. (A) Statistical analysis of GGT1 mRNA in PC12 cells treated with EA or untreated after OGD modeling (n = 3). (B) Western blot (WB) was used to detect GGT1 protein expression after CHX treatment. (C) GGT1 protein stability was assessed after EA or DMSO treatment under temperature gradient conditions. (D) Quantitatively analyze the grayscale values of GGT1 and plot the thermal melting curve of GGT1. (E) The real‐time response curve of GGT1 interacting with a series of EA concentrations (25–5000 ug/mL) shows an increasing binding platform response as EA concentration rises. (F) The protein expression of GGT1 in PC12 cells was detected by Western blot after treatment with proteasome inhibitor MG132 and lysosome inhibitor CQ. (G) Quantitative analysis of GGT1 expression (n = 3). (H) Representative SPR plots of GGT1 and MGRN1 binding kinetics, showing the fact response curves for immobilized GGT1 bound to 5 uM MGRN1. The red curve represents the response curve with 0.5 mg/mL EA added, while the blue curve represents the response curve with DMSO added. (I) Molecular docking of GGT1 with MGRN1. (J) Sequence alignment of Ub loci in GGT1 histogram of different species. (K) HEK293T cells were transfected with truncated GGT1 lysine mutant (GGT1‐K2A) and MGRN1 for WB. (L) Quantitative analysis of GGT1 and MGRN1 expression (n = 3). (M, N) WB analyses of ubiquitination of GGT1 or GGT1 K2A mutant in MGRN1 overexpressed HEK‐293T cells. (O) HEK‐293T cell lysates transfected with various HA‐tagged ubiquitin mutants (including K6, K11, K29, K33, K48, and K63) and corresponding overexpressed plasmids were treated with MG132 before harvest to obtain anti‐Flag antibody IP, and then ubiquitin was detected by WB. (P, Q) HEK‐293T cell lysates and corresponding overexpressed plasmids were transfected with HA‐tagged Ubi or HA‐tagged Ubi K48R, IP with anti‐FLAG antibody, and then the ubiquitin of GGT1 was detected with anti‐HA antibody. Data are expressed as mean ± SD, All are comparisons between each group and control, *: *p* < 0.05, **: *p* < 0.01 and ***: *p* < 0.001.

To elucidate the mechanism underlying EA‐mediated suppression of GGT1 protein levels, we first examined its effect on transcription. Q‐PCR revealed that EA did not significantly reduce GGT1 mRNA expression (Figure 4A), suggesting that its regulatory action involves post‐translational modification (PTM). We therefore investigated whether EA promotes degradation of the GGT1 protein. Using cycloheximide (CHX) to halt new protein synthesis, we observed that EA markedly accelerated the decay of existing GGT1 protein (Figure 4B).

To assess potential direct interactions, we performed a cellular thermal shift assay (CETSA), which detects ligand‐target binding through thermal stabilization. EA treatment caused a rightward shift in the GGT1 thermal denaturation curve, indicating that EA directly interacts with GGT1 and enhances its thermal stability. This suggests a physical interaction between EA and GGT1, which may induce conformational changes that facilitate subsequent recognition by the E3 ligase MGRN1 (Figure 4C,D). Surface plasmon resonance (SPR) results indicate that the physical binding of EA to GGT1 exhibits concentration dependence (Figure 4E).

We sought to identify the relevant degradation pathway. Treatment with the proteasome inhibitor MG132, but not the lysosomal inhibitor chloroquine (CQ), effectively blocked EA‐induced reduction of GGT1 protein. Taken together, these data indicate that EA promotes GGT1 degradation primarily through the ubiquitin‐proteasome pathway (Figure 4F,G). To identify the specific E3 ligase mediating GGT1 ubiquitination, we employed immunoprecipitation‐mass spectrometry (IP‐MS) analysis, which identified MGRN1 as a key candidate (Figure S6A–E). Kinetic analysis revealed that EA increased the association rate constant (Ka) from 0.11 × 10^3^ M^−^^1^s^−^^1^ (DMSO group) to 3.98 × 10^3^ M^−^^1^s^−^^1^ (EA group), while reducing the dissociation rate constant (Kd) from 0.097 to 0.016 s^−^^1^. Collectively, EA achieved approximately 217‐fold enhancement in the equilibrium binding affinity between GGT1 and MRGN1 (Figure 4H). Molecular docking experiments further confirmed the high‐affinity interaction between GGT1 and MGRN1 (Figure 4I). MGRN1 is a RING‐finger E3 ubiquitin ligase with well‐defined functions in neurodevelopment, synaptogenesis, and neural signal transduction.

**FIGURE 5:**
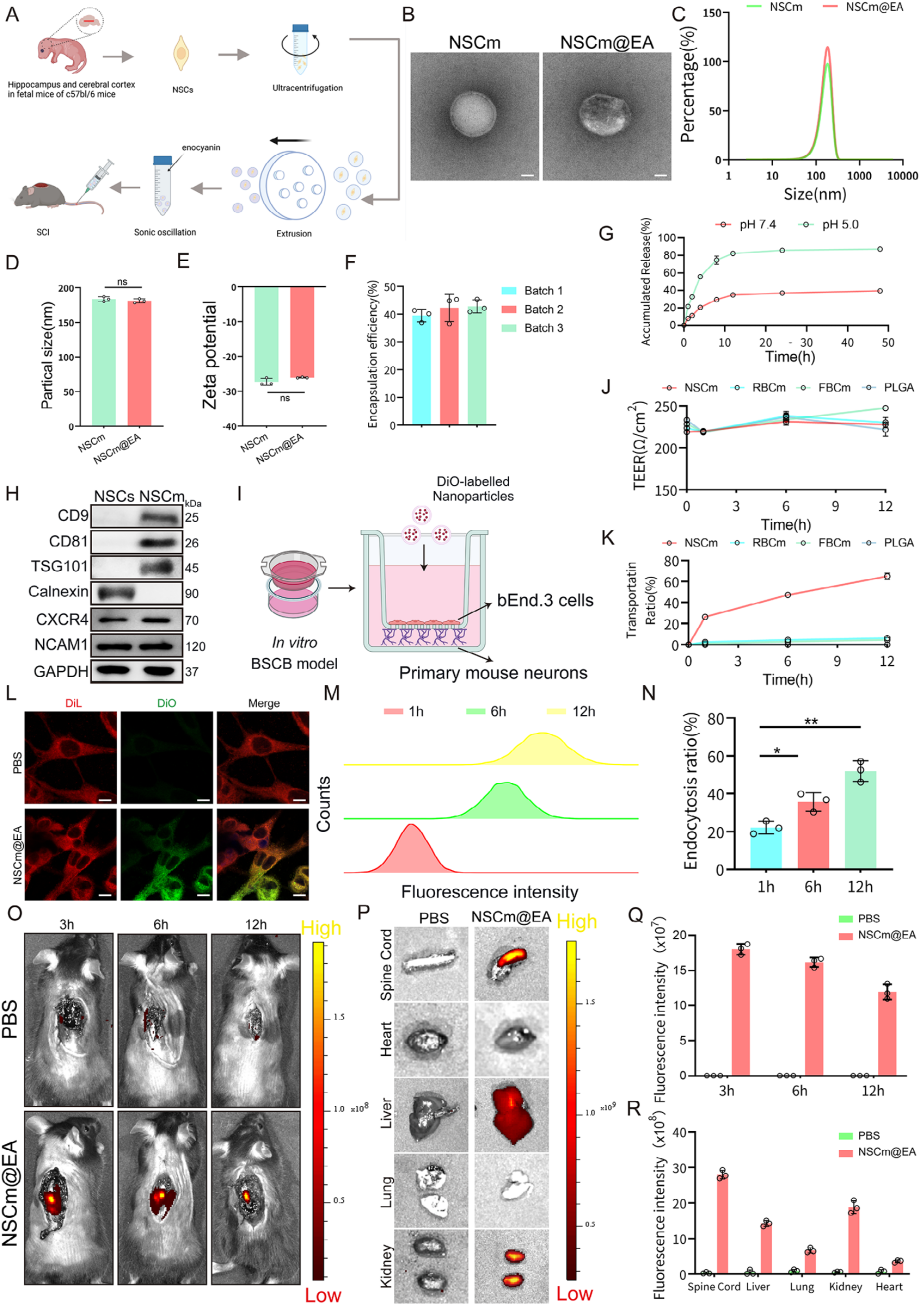
Construction and Characterization of NSCm@EA. (A) Schematic diagram of NSCm preparation, separation, and drug loading. (B) Morphology of NSCm and NSCm@EA under TEM. (C) NTA analysis showed NSCm and NSCm@EA particle size distribution. (D,E) Quantitative analysis of particle size and Zeta potential for NSCm and NSCm@EA (n = 3). (F) Encapsulation efficiency of NSCm@EA prepared from three independent batches, demonstrating reproducible and satisfactory encapsulation performance.(n = 3) (G) Cumulative release curve of EA in PBS at pH 7.4 and pH 5.0 over 48 h for NSCm@EA. (n = 3) (H) WB was used to detect the expression levels of NSCm biomarkers CD9, CD81, and TSG101 as well as CXCR4, NCAM1, and negative control Calnexin protein. (I) Schematic illustration of the in vitro blood‐spinal cord barrier (BSCB) model. (J) Comparison of transendothelial electrical resistance (TEER) values between the upper and lower chambers, confirming the integrity of the barrier. (K) Proportion of NSCm, RBCm, FBCm, and PLGA nanoparticles that migrated across the in vitro BSCB model. (L) Using DiL label PC12 cell membranes, DiO label NSCm@EA. The colocalization of DiL fluorescence and DiO fluorescence was observed under a laser confocal microscope. (M,N) Flow cytometry revealed the uptake rates of neurons for NSCm@EA at different time points and their statistical analysis. (O,Q) Representative images of NSCm@EA accumulation in the body after intratumoral injection via the tail vein within a specified time period (n = 3). (P, R) In vitro imaging shows the distribution of PBS and NSCm@EA in the isolated spinal cord and viscera (n = 3). Data are expressed as mean ± SD, All are comparisons between each group and control, *: *p* < 0.05, **: *p* < 0.01, and ***: *p* < 0.001.

Using the UniProt database for site prediction alongside our docking results, we identified a single lysine residue (K2) on GGT1 as the probable ubiquitination site. This residue is notably conserved across species (Figure 5J). To test its functional relevance, we generated a point mutant where lysine was substituted with alanine (K2A). Unlike wild‐type (WT) GGT1, the K2A mutant was resistant to MGRN1 overexpression‐mediated degradation (Figure 5K,L). This resistance was due to significantly reduced ubiquitin binding in the K2A mutant, confirming the critical role of the K2 site in MGRN1‐mediated ubiquitination (Figure 5M,N). We then screened the topology of the ubiquitin chain attached to GGT1. Immunoblotting with linkage‐specific antibodies revealed prominent ubiquitination via K48‐linked chains, whereas signals for K6‐, K11‐, K29‐, and K63‐linked chains were undetectable (Figure 5O). To corroborate this specificity, we co‐transfected HEK 293T cells with GGT1 and either HA‐tagged WT ubiquitin or a K48R mutant that cannot form K48 linkages. Co‐immunoprecipitation (Co‐IP) demonstrated robust GGT1 ubiquitination in the presence of WT ubiquitin, which was drastically diminished with the K48R mutant (Figure 5P,Q). This confirmed that GGT1 is selectively modified by K48‐linked polyubiquitin chains.

Collectively, these findings establish that MGRN1 mediates K48‐linked polyubiquitination of GGT1 at the evolutionarily conserved K2 residue, targeting it for proteasomal degradation, while EA enhances this process.

### 2.5 Construction and Characterization of NSCm@EAs from Neural Stem Cells

To address the limitations of EA translation: its inherent instability and low potency resulting in insufficient bioavailability, we sought to develop strategies to overcome these obstacles and enhance its therapeutic efficacy.

We extracted NSCs membranes to serve as a biomimetic carrier for EA (Figure 5A). Both empty NSC membranes (NSCm) and EA‐loaded vesicles (NSCm@EA) exhibited a characteristic lipid bilayer structure under scanning electron microscopy (Figure 5B). The encapsulation of EA did not alter the vesicle size, with both formulations maintaining a diameter of approximately 180 nm (Figure 5C,D). Similarly, since we loaded EA internally without modifying the membrane surface, the Zeta potentials of NSCm and NSCm@EA showed no significant difference (Figure 5E). The encapsulation efficiency of three independent batches was 41.5% ± 2.3%, indicating reproducibility of our surface preparation process (Figure 5F). Release profiles of NSCm@EA were evaluated in PBS at pH 7.4 (physiological) and pH 5.0 (lysosomal). At pH 7.4, 34.83% of EA was released within 12 h, whereas 82.29% was released under acidic conditions (Figure 5G). Western blot analysis confirmed the enrichment of characteristic membrane markers CD9, CD81, and TSG101, as well as CXCR4, NCAM1 in NSCm, while the endoplasmic reticulum marker calnexin was absent, indicating successful membrane isolation with minimal cytoplasmic contamination (Figure 5H).

We established an in vitro BSCB model based on brain endothelial cells (bEnd.3 cells) and compared NSCm with other nanocarriers, including erythrocyte membranes (RBCm), fibroblast membrane (FBCm), and PLGA (Figure 5I). During a 12‐h incubation, TEER values remained stable across all groups, indicating maintained structural integrity of the BSCB (Figure 5J). Notably, only NSCm showed efficient and sustained translocation across the intact barrier, whereas the other nanoparticles exhibited minimal permeability (Figure 5K). To evaluate targeting capability in vitro, DiO‐labeled NSCm demonstrated significant fluorescence co‐localization with DiL‐labeled PC12 cell membranes, confirming effective neuronal binding (Figure 5L). Furthermore, NSCm can gradually cross the BSCB over time and accumulate progressively within neurons (Figure 5M,N).

We next assessed the in vivo distribution of Dil‐labeled NSCm@EA using an in vivo imaging system (IVIS) (Figure 5O). NSCm@EA effectively accumulated at the spinal cord injury site, retaining 66.3% of its initial fluorescence intensity 12 h post‐injection (Figure 5Q). Ex vivo imaging of isolated organs further revealed high enrichment of the signal in the injured spinal cord, with clearance primarily through the liver and kidneys (Figure 5P,R).

In summary, the NSC membrane‐based delivery platform successfully achieves preferential accumulation at the spinal cord lesion in a mouse model, offering a promising strategy to significantly improve the bioavailability and therapeutic potential of EA.

### 2.6 NSCm@EA Promotes the Motor Function Recovery and Axonal Regeneration in SCI Mice

We first evaluated the axonal regeneration‐promoting efficacy of NSCm@EA in vitro. Treatment with NSCm@EA significantly increased the number of regenerating neurons co‐labeled with MAP2 and NeuN, although this effect did not differ significantly from that of EA alone (Figure S7A,B). Correspondingly, both NSCm@EA and EA effectively reduced ROS levels and helped preserve mitochondrial structure, with no notable difference between the two treatments (Figure S7C–E). However, we observed that NSCm@EA more potently enhanced neuronal autophagy compared to EA alone, contributing to its neuroprotective effect (Figure S7F,G).

**FIGURE 6:**
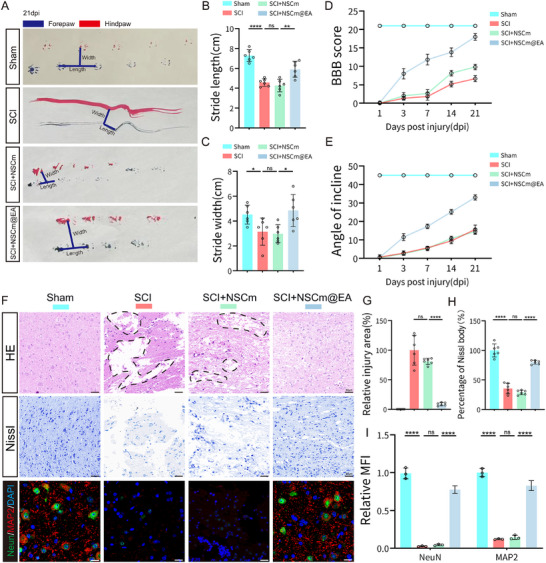
Evaluation of NSCm@EAs treatment efficacy in vivo. (A) Analysis of gait with representative footprints at 21 days after SCI. (B,C) Statistical Analysis of Stride Length and Stride Width (n = 6). (D,E) BBB scores and oblique plate Angle at 1,3,7,14, and 21 days after SCI (n = 6). (F) HE staining and Nissl staining of spinal cord longitudinal sections of mice 21 days after sci. Scale bars: 50 µm. IF was used to detect the immunoreactivity of Neun (green‐CoraLite488) and MAP2 (red‐CoraLite594). Scale bars: 50 µm. (G,H) Statistical analysis of HE and Nissl staining (n = 6). (I) Quantitative analysis of fluorescence intensity of NeuN and MAP2 (n = 3). Data are expressed as mean ± SD, *: *p* < 0.05, **: *p* < 0.01, and ***: *p* < 0.001.

Encouraged by these in vitro results, we next assessed the therapeutic utility of this strategy in vivo. By day 21 post‐injury, NSCm@EA effectively restored the reductions in stride length and width caused by SCI (Figure 6A–C), improved the BBB score to 18, and increased the climbing angle to 33°— gains nearly double those achieved with EA treatment alone (Figure 6D,E). These findings demonstrate that the delivery strategy substantially enhances the bioavailability of EA and markedly improves motor function. Furthermore, NSCm@EA reduced the spinal lesion area by 92.6% and increased the proportion of Nissl bodies within neurons by 40.7%, outcomes that were not attained with NSCm alone (Figure 6F–H). Finally, NSCm@EA also exhibited a robust therapeutic effect in upregulating the expression of neuronal and axonal regeneration marker proteins (Figure 6F,I).

### 2.7 NSC‐m@EA Elicits Predominantly Neuron‐Specific Protective Effects with Minimal Impact on the Global Spinal Cord Milieu Landscape in SCI: A Single‐Cell Atlas

**FIGURE 7:**
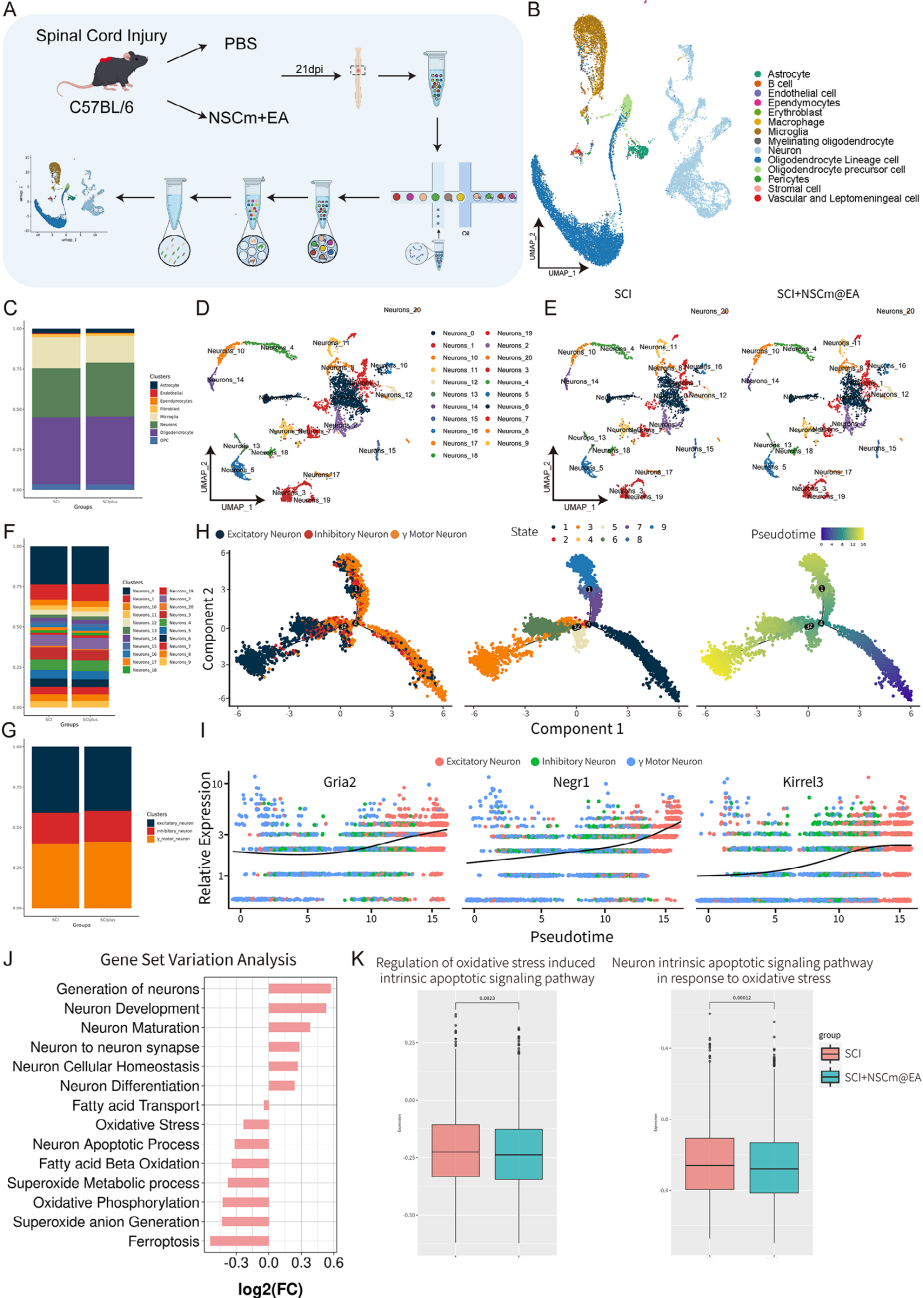
Underlying Mechanism of NSCm@EA‐Mediated Neural Repair in SCI at Single‐Cell Resolution. (A) Schematic overview of the single‐cell RNA sequencing (snRNA‐seq) experimental workflow. Mice subjected to spinal cord injury (SCI) were treated with either PBS (SCI group) or NSCm@EA (SCI + NSCm@EA group) for 21 days prior to analysis. (B) UMAP projection of the cellular landscape in the spinal cord, comparing the PBS‐treated (SCI) and NSCm@EA‐treated (SCI + NSCm@EA) groups. Colors represent distinct cell clusters. (C) The proportions of major cell populations in each group. (D) UMAP projection of the neuronal subclusters. (D) UMAP plots focusing specifically on neuronal subclusters. Colors represent distinct cell clusters. (E) UMAP projection of neuronal cell subpopulations in SCI and SCI + NSCm@EA group respectively. (F) The bar chart quantifies the proportional changes in neuronal subtypes following treatment. (G) The ratio of excitatory sensory neurons, inhibitory sensory neurons, and γ‐motor neurons in the spinal cord between two groups. (H) Pseudotime trajectory analysis depicting the inferred differentiation paths of excitatory sensory neurons, inhibitory sensory neurons, and γ‐motor neurons within the spinal cord. (I) Dynamic expression patterns of gene sets associated with neuronal and axonal regeneration across neuronal subpopulations. (J) Bar chart of GSVA‐enriched differentially expressed genes in excitatory and inhibitory neurons between the SCI group and the SCI + NSCm@EA group. Terms are ranked in descending order based on the absolute log2(FC) value. (K) Boxplots show the GSVA terms associated with neuronal intrinsic apoptosis.

To examine the neuron‐specific neuroprotective effects of NSCm@EA following spinal cord injury, we performed single‐nucleus RNA sequencing on spinal cord tissues harvested 21 days post‐injury from mice treated with either PBS or NSCm@EA (Figure 7A). After filtering out low‐quality cells and potential doublets, 10 227 high‐quality cells were retained for analysis. Based on the top six differentially expressed genes per cluster, we identified 14 distinct cell types, including astrocytes, B cells, ependymocytes, erythroblasts, macrophages, microglia, myelinating oligodendrocytes, neurons, oligodendrocyte lineage cells, oligodendrocyte precursor cells (OPCs), pericytes, stromal cells, and vascular and leptomeningeal cells (Figure 7B; Figure S8A).

Analysis of cellular proportions revealed that NSCm@EA treatment specifically increased the percentage of neurons without altering the composition of other major cell types (Figure 7C). Given the central role of neurons in functional recovery, we next investigated how NSCm@EA reshapes neuronal heterogeneity after injury. Using established cell markers, we subdivided all neurons into 21 transcriptionally distinct subclusters (Neuron 0–20), visualized separately for each treatment group (Figure 7D,E). In the NSCm@EA group, the proportion of neuron 1 was significantly increased while correspondingly reducing the proportion of neuron 3, indicating that the treatment induced a state transition in a subset of neurons (Figure 7F). To further explore this transition, we re‐annotated the neuronal subsets into three functional classes: excitatory neurons, inhibitory neurons, and γ‐motor neurons (Figure S8B). NSCm@EA increased the proportion of γ‐motor neurons while decreasing that of excitatory neurons, with no significant change in inhibitory neurons (Figure 7G). Since γ‐motor neurons fine‐tune muscle spindle sensitivity, their expansion likely contributes to the improved motor coordination observed in treated mice. Conversely, the reduction in excitatory neurons may alleviate neuropathic pain and spasticity. Together, these shifts suggest that NSCm@EA induces a profound, functionally coherent remodeling of the neuronal landscape.

To investigate whether this remodeling involves changes in cellular identity, we performed pseudotime trajectory analysis. A subset of sensory neuron‐like cells exhibited a transcriptional shift toward a motor neuron‐like signature—an unexpected convergence that likely reflects therapy‐induced reprogramming of the transcriptional landscape toward a unified, pro‐repair state. These cells clustered closer to γ‐motor neurons (Figure 7H). Along this trajectory, synaptic genes such as *Gria2*, *Negr1*, and *Kirrel3* were coordinately upregulated, indicating active synaptic reassembly and functional maturation of neurons under NSCm@EA treatment (Figure 7I). Gene set variation analysis (GSVA) of differentially expressed genes across neuronal subsets further demonstrated that NSCm@EA promotes neurogenic processes while suppressing ferroptosis, oxidative stress, and ultimately, neuronal apoptosis (Figure 7J,K; Figure S8C).

In summary, single‐nucleus transcriptomics reveals that NSCm@EA mediates neural repair not through indiscriminate neurogenesis, but by selectively remodeling specific neuronal subpopulations, steering the injured spinal cord toward a functional, pro‐recovery cellular architecture.

## 3 Discussion

Spinal cord injury (SCI) remains a devastating condition with high mortality and morbidity [31, 32], where the complexity of its pathophysiology presents major barriers to neuronal regeneration and functional recovery, underscoring the urgent need for effective treatments [33]. This study systematically elucidates the central role of GGT1 in driving neuronal ferroptosis after SCI and establishes a complete translational pipeline—from target discovery and lead compound identification to the development of a biomimetic delivery system. Our work not only clarifies the unique mechanism by which the natural product EA exerts neuroprotection by promoting GGT1 ubiquitination and degradation, but also introduces an innovative NSCm@EA nanodelivery system that uses neural stem cell membrane coating. This strategy overcomes the inherent limitations of EA, significantly enhancing its bioavailability and targeting specificity both in vitro and in vivo, and achieving superior motor recovery and neuronal repair compared to free EA.

While acute primary mechanical injury can be alleviated by emergency surgery [34], secondary neuronal death during the acute/subacute phase often dictates functional outcomes [35]. We identifie GGT1 as a critical checkpoint regulating neuronal fate after SCI. Unlike prior studies focused on core ferroptosis execution proteins such as GPX4 and SLC7A11, we target GGT1, an upstream metabolic regulator. By hydrolyzing glutathione (GSH), GGT1 disrupts cellular redox homeostasis and directly promotes lipid peroxidation and ferroptosis. This finding tightly links antioxidant reserve metabolism to ferroptotic execution, expanding our understanding of secondary injury mechanisms.

The neuroprotective benefits of inhibiting ferroptosis after SCI have been consistently demonstrated across multiple independent studies, with this intervention being linked to improved functional outcomes [36, 37, 38, 39]. Our finding that GGT1 acts as a key metabolic node influencing the cellular redox state further supports the emerging view that metabolic reprogramming plays a crucial role in determining neuronal susceptibility to ferroptosis. Together, these converging insights reinforce the rationale for targeting ferroptosis as a therapeutic strategy in SCI.

The lead compound EA identified here operates via a distinct mechanism. Most reported ferroptosis inhibitors function by scavenging lipid radicals or suppressing lipid peroxidation directly. In contrast, EA promotes K48‐linked polyubiquitination and subsequent proteasomal degradation of GGT1, reducing the accumulation of this pro‐death protein at its source. This mechanism aligns with the emerging paradigm of targeted protein degradation, offering a novel strategy for neuroprotection [40, 41, 42]. As a single‐component natural molecule, EA also presents practical advantages, including synthetic accessibility and relatively well‐defined metabolism. Further transcriptomic and functional validation revealed that the EA/GGT1 axis activates the PI3K/AKT/mTOR pathway. Notably, EA partially restored PI3K/AKT/mTOR signaling even in the presence of the PI3K inhibitor 3‐MA. This apparent paradox may reflect incomplete PI3K inhibition under our conditions, along with PI3K‐independent effects of EA. For example, reduced oxidative stress may relieve PTEN‐mediated suppression of AKT, while improved mitochondrial function may enhance mTOR activity via energy‐sensing pathways such as AMPK. Together, these findings suggest that EA exerts neuroprotection through both PI3K‐dependent and independent mechanisms.

While previous studies have linked glutathione (GSH) metabolism to ferroptosis, most have focused on its synthesis or the role of GPX4 [43, 44, 45]. Our study takes a different approach by identifying GGT1—a degradative enzyme—as a direct target. We further demonstrate that the natural compound EA eliminates GGT1 through the ubiquitin‐proteasome pathway, a mechanism distinct from the direct antioxidant actions of most known ferroptosis inhibitors. The mechanism by which this small molecule induces ubiquitin‐mediated protein degradation provides an emerging paradigm for the development of neuroprotective drugs. EA is analogous to natural molecular glue; it induces GGT1 ubiquitination and degradation by enhancing the GGT1‐MGRN1 interaction. However, it should be specially noted that EA is only a natural small molecule, significantly different from Proteolysis Targeting Chimera (PROTAC). It is important to note that GGT1 has been shown to exert protective effects in certain chronic neurodegenerative or cancer contexts, often by helping maintain glutathione levels [46, 47, 48]. This apparent discrepancy likely arises from differences in disease context, cell type, and the pathological stage. During the acute secondary injury phase of SCI, however, intense oxidative stress rapidly depletes GSH [49, 50]. In this environment, highly active GGT1 may exacerbate oxidative damage by depleting the already limited glutathione reserve. Our data support a role for GGT1 in promoting injury in this specific spatiotemporal context—namely, in neurons during acute injury. This highlights the complexity of pathological mechanisms, where the same molecule can have opposing effects depending on the microenvironment or stage of disease. While definitive proof would require gain‐of‐function studies, the existing loss‐of‐function and pharmacological data provide a robust foundation for GGT1 as a therapeutic target in SCI. This study is a proof‐of‐concept, focusing on advancing the understanding of ferroptosis mechanisms in SCI and integrating targeted protein degradation with bionic nanomedicine applications in neural repair, thereby providing a solid experimental foundation for SCI repair.

Despite its promising activity, EA's inherent instability and low bioavailability limit its therapeutic application. To address these long‐standing challenge in central nervous system drug delivery, we developed an innovative strategy. Although the targeting efficiency and biocompatibility of various synthetic nanocarriers (e.g., liposomes [51, 52]) remain suboptimal, the engineered system framework can serve as a reference, providing critical insights for optimizing the release characteristics and targeting efficiency of NSCm@EA in subsequent applications. Inspired by the injury‐interacting properties of neural stem cells [53], our NSCm@EA system ingeniously combines the biological functionality of biological membranes with the drug‐loading capacity of nanoparticles. Single‐cell sequencing and behavioral analyses confirm that this system not only associates with improved neuronal outcomes at the injury site but also achieves precise neuronal repair without disrupting the broader microenvironment—such as microglial phenotypes or astrocytic responses—marking a significant advance in therapeutic precision.

While this study provides substantial advances, several limitations warrant consideration. First, the observed accumulation of NSCm@EA at the spinal cord injury site may be partially attributed to the passive EPR effect induced by BSCB disruption. Distinguishing between passive targeting and active targeting is crucial for the rational design of nanodelivery systems [54]. Second, long‐term efficacy and safety need comprehensive evaluation. This work primarily focused on acute to subacute outcomes; the chronic phase of SCI involves neural remodeling, glial scar maturation, and circuit reorganization. Assessing NSCm@EA in chronic injury models and systematically studying its biodistribution, clearance, and potential immunogenicity will be critical for clinical translation. Third, practical challenges in clinical translation remain, particularly the scalable and standardized production of NSCm.

## 4 Conclusions

In summary, this multidimensional study provide evidence that targeting GGT1 for ubiquitin‐mediated degradation represents a novel and effective strategy for SCI. The natural compound EA serves as the effector of this mechanism, and its integration with the biomimetic NSCm delivery system createes a potent and precise therapeutic platform. By advancing the understanding of ferroptosis in SCI and converging the fields of targeted protein degradation and biomimetic nanomedicine for neural repair, this work provides a solid experimental foundation and innovative proof of concept.

## 5 Experimental Section

### 5.1 Animals

This study involved an experiment on male C57BL/6 mice weighing 20 – 30 g, sourced from Hangzhou Resources Laboratory Animal Technology Co., Ltd. in Hangzhou, China (with a license number SCXK 2019 – 0004). The experimental procedures strictly adhered to the Guide for the Care and Use of Laboratory Animals of the Chinese Institutes of Health and were approved by the Animal Ethics Committee of the Laboratory Animal Center of the First Affiliated Hospital of the University of Science and Technology of China (approval number 2024 – N (A) – 134). The mice were housed individually in standard experimental cages under a 12 – h light/dark cycle and had access to food and water ad libitum.

### 5.2 Animal Models of Spinal Cord Injury and Treatment

This study investigated the effects of a specific spinal cord injury in mice. To ensure pain—free procedures, mice were anesthetized with sevoflurane (initiated at a 3% concentration and maintained at 1%) and kept on a temperature—controlled blanket to maintain a body temperature of 37°C. A laminectomy was performed to expose the spinal cord, followed by a weight—drop injury model where a 15 g iron bar was dropped from a height of 15 mm to induce T10 moderate contusion [55, 56]. After the injury, the mice were sutured and required post—operative care, including three times—a—day bladder emptying due to limited mobility. A control group of mice underwent the same surgery but without the injury, providing a basis for comparison and enabling a controlled study of the spinal cord injury effects, with careful surgical and post—operative management to ensure accurate results. Mice were assigned to the corresponding groups. The sham group underwent only a laminectomy, while the other groups additionally received SCI modeling and corresponding therapeutic interventions.

### 5.3 Basso–Beattie–Bresnahan Score

The Basso—Beattie—Bresnahan (BBB) scale [57], ranging from 0 (no movement at all) to 21 (normal movement like healthy mice), was employed to quantify their functional recovery. Scores were recorded daily, and comparisons were made among different groups of mice at 1 day, 3 days, 7 days, 14 days, and 21 days post—injury.

### 5.4 Footprint Analysis

Mice's hind limbs were dipped in red and blue ink, and they were allowed to walk on a white track, leaving distinct footprints. The scientists meticulously recorded the paths and measured two critical parameters: stride width and stride length. Notably, the testers were blinded to the specific groups of mice being examined, ensuring unbiased and objective results.

### 5.5 Ramp Test

Mice were placed in the test device, a ramp covered with a 3‐mm thick rubber mat. The bodies of the mice were positioned parallel to the longitudinal axis of the inclined plate. The mice started from the bottom of the ramp and gradually ascended at a 5‐degree angle for 5 s. Each animal underwent three trials, and the average value was used as the measured result.

### 5.6 Transcriptome Sequencing and Bioinformatics Analyses

Transcriptome analysis was performed on the OGD and Enocyanin+OGD groups (n = 3/group). In the OGD group, cells were subjected to pure OGD‐induced cell death for 4 h and then collected. In the Enocyanin + OGD group, the cells were treated with Enocyanin for 2 h before being exposed to OGD for 4 h. Total RNA was extracted using TRIzol reagent according to the manufacturer’ specifications. RNA purity and quantity were determined using a NanoDrop 2000 spectrophotometer (Thermo Scientific, USA), and RNA integrity was assessed with an Agilent 2100 Bioanalyzer (Agilent Technologies, Santa Clara, CA, USA). Transcriptome libraries were constructed using the VAHTS Universal V6 RNA‐seq Library Prep Kit following the manufacturer's instructions. Transcriptome sequencing and analysis were conducted by Shanghai Ouyi Biological Technology Co., Ltd. (Shanghai, China).

### 5.7 Cell Culture

The PC12 neuron cell line (Cat# CL‐0481, RRID: CVCL_0481) was obtained from Procell Life Science and Technology Co., Ltd. (Wuhan). Primary neural stem cells were isolated as previously described [58, 59]. The cells were cultured in a humidified atmosphere with 5% CO2 at 37°C in Dulbecco's modified Eagle's medium (DMEM, Procell, Wuhan, China, Cat# 319‐005‐CL) supplemented with glucose, 10% fetal bovine serum (Corning, NY, USA, Cat# 35‐881‐CV), 100 mg/mL streptomycin and 100 U/mL penicillin‒streptomycin‐amphotericin B solution (100X, Beyond Times, Shanghai, China, Cat# C0224‐100 mL).

### 5.8 Preparation of NSCm and NSCm@Enocyanin

We use PBS to prepare a 1 mL cell suspension of 2 × 10^6 NSCs cells/mL. Sonicate the cell suspension briefly: 40% power, 2 s on, 2 s off, for a total of 0.5 min. Centrifuge at 4°C for 10 min to remove nuclei and unbroken cells; mix enocyanin with the supernatant obtained after centrifugation and load the enocyanin into the NSCm by ultrafugation; obtain NSCm@Enocyanin by continuous extrusion through a polycarbonate filter membrane with a pore size of 100 nm.

### 5.9 Determination of Encapsulation Efficiency and In Vitro Release

Encapsulation efficiency (EE%): Free Enocyanin was separated from NSCm@EA by ultracentrifugation (100 000 × g, 4°C, 1 h). Drug content in the supernatant and pellet was quantified by HPLC. EE% = (total drug – free drug) / total drug × 100%.

### 5.10 In Vitro Release

NSCm@EA suspension (1 mL, containing 100 µg Enocyanin) was sealed in a dialysis bag (10 kDa MWCO) and immersed in release medium (PBS with 0.5% Tween‑80, pH 7.4; or acetate buffer with 0.5% Tween‐80, pH 5.5) at 37°C with shaking at 100 rpm. At designated time points (0.5–72 h), aliquots were withdrawn and analyzed by HPLC to calculate cumulative release. All measurements were performed in triplicate.

### 5.11 Oxygen Glucose Deprivation (OGD) Cell Model

PC12 cells were cultured in glucose‐free deoxygenated DMEM (Gibco, 11966025) and subjected to oxygen‒glucose deprivation by incuation in a hypoxia chamber (Thermo Fisher Scientific, Waltham, MA, USA) at 37°C for 4 h. Control cells were cultured under normoxic conditions (95% O_2_ and 5% CO_2_ and N_2_) in DMEM and 10% FBS for the same duration. The cells were pretreated with enocyanin (0.5 mg/mL), 3‐MA (10 mM), or CQ (30 µM) for 2 h before exposure to OGD, followed by autophagy assays.

### 5.12 Cell Viability Assessment

PC12 cells were seeded into 96‐well plates and pretreated with Enocyanin (0 to 8 mg/mL) or H2O2 (0–256 mM) at 37°C for 24 h. Following this, 10 µL of Cell Counting Kit‐8 solution (Beyond Times, Shanghai, China, Cat# C0038) was added to each well, and the cells were incubated for an additional 30 min at 37°C. Absorbance values were then measured at 450 nm using a microplate reader (Thermo Fisher Scientific Inc., Multiskan MK3).

### 5.13 Western Blotting

Total protein was extracted from PC12 cells, primary neural stem cells, neural stem cell membrane vesicles, or spinal cord tissue and then separated by sodium dodecyl sulfate‒polyacrylamide gel electrophoresis. The proteins were then transferred to a polyvinylidene fluoride membrane (Millipore, Billerica, MA, USA)and blocked with room temperature blocking buffer (Epizyme, Shanghai, China) for 1 h. The membranes were subsequently incubated overnight at 4°C with the following primary antibodies: anti‐γ‐glutamyltransferase 1 (GGT1) (1:1000, Affinity, Cat#DF6610, RRID:AB_2838572), anti‐microtubule‐binding protein 2 (MAP2) (1:500, Proteintech, Cat#17490‐1‐AP, RRID: AB_2137880), anti‐neurofilament protein‐200 (NF200) (1:5000, Proteintech Cat# 60331‐1‐IG, RRID:AB_2881440), anti‐stathmin2 (STMN2) (1:2000, Proteintech, Cat#67204‐1‐IG, RRID:AB_2882497), anti‐coiled‐coil, moesin‐like BCL2‐interacting protein (Beclin‐1) (1:1000, Proteintech, Cat#1306‐1‐AP, RRID:AB_2259061), anti‐P62/Sequestosome‐1 (SQSTM/p62) (1:500, Affinity, Cat#AF5384, RRID:AB_2837869), anti‐microtubule‐associated protein 1 light chain 3 (LC3) (1:1000, Proteintech, Cat#14600‐1‐AP, RRID:AB_2137737), anti‐phosphatidyl inositol 3 kinase (PI3K) (1:1000, Cell Signaling Technology, Cat#4257, RRID: AB_659889), anti‐phosphorylated phosphatidyl inositol 3‐kinase (p‐PI3K) (1:1000, Affinity, Cat#AF3242; RRID:28 34668), anti‐protein kinase B (AKT) (1:1000, Cell Signaling Technology, Cat#4691, RRID:AB_915783), anti‐phosphorylated protein kinase B (p—AKT) (1:1000, Cell Signaling Technology, Cat#9271, RRID: AB_329825), anti‐mammalian target of rapamycin (mTOR), (1:1000, Cell Signaling Technology, Cat#2983, RRID:AB_2105622), anti‐protein phosphorylation of mammalian target of rapamycin (p‐mTOR) (1:1000, Cell Signaling Technology, Cat# 5536, RRID:AB_10691552), anti‐CD81 (1:500, Affinity, Cat#DF2306, RRID:AB_2839530), anti‐CD9 (1:500,Affinity,Cat#AF5139,RRID:AB_2837625), anti‐tumor susceptibility (TSG101) (1:500, Affinity, Cat#DF8427, RRID:AB_2841675), anti‐Calnexin (1:500, Affinity, Cat#AF5362, RRID:AB_2837847) and anti‐glyceraldehyde‐3‐phosphate dehydrogenase (GAPDH) (1:50 000, Proteintech,Cat#60004‐1‐IG, RRID:AB_2107436).

The following day, the membranes were incubated at 25°C for 2 h with secondary antibodies conjugated with horseradish peroxidase, including HRP‐conjugated affinity‐purified goat anti‐mouse IgG (H + L) (1:50,000 dilution, Proteintech, Cat# SA00001‐1, RRID: AB‐2722565) and HRP‐conjugated affinity‐purified goat anti‐rabbit IgG (H+L) (1:50 000 dilution, Proteintech, Cat# SA00001‐2, RRID: AB‐2722564). Protein bands were visualized using enhanced chemiluminescence (ECL) reagent (KF8003, Affinity, Liyang, Jiangsu Province, China). Images were captured using a chemiluminescence gel imaging system (Proteinsimple, FluorChem R, San Francisco, CA, USA). Image analysis was performed using ImageJ software (ImageJ2, National Institutes of Health, Bethesda, MD, USA; Schneider et al., 2012). Allexperiments were conducted in triplicate, and the results were normalized to those of the blank group.

### 5.14 Short Hairpin RNARNA (shRNA) Transfection

ShRNAs were designed and synthesized by Anhui General Biological Products Co., Ltd., located in Chuzhou, Anhui, China. The transfer solution was prepared by diluting 5 µL of Lipo 3000 (Invitrogen, Carlsbad, CA, USA, Cat# L3000001) in 125 µL of Opti‐MEM (Invitrogen, Cat# 31985070). Simultaneously, 5 µL of shRNA and 10 µL of p3000 were diluted together in a separate 125 µL of Opti‐MEM. The two solutions were mixed, incubated at 37°C for 15 min, and then added to the PC12 cells in six‐well dishes. The knockdown efficiency of the shRNAs was evaluated using quantitative polymerase chain reaction (RT‒qPCR) and Western blotting.

### 5.15 RT‒qPCR

TRIzol reagent (Life Sciences, Shanghai, China) was used to isolate total RNA from PC12 cells following the manufacturer's instructions,. RNA concentration was determined using a NanoDrop spectrophotometer (Thermo Fisher Scientific, Waltham, MA, USA). The HiScript II Q RT SuperMix reagent kit (Vazyme, Cat# R122‐01, China) was used to reverse transcribe RNA into cDNA, which served as a template for quantitative polymerase chain reaction (RT‒PCR). RT‐PCR was performed using AceQ‐qPCR SYBR Green Master Mix (Vazyme, Cat# Q111‐02) and a real‐time fluorescent quantitative PCR system (Roche, Basel, Switzerland, LightCycler480 II).Primers were used to detect GGT1, MAP2, STMN2, NF200, GAP43, β‐tubulin, LC3, Beclin‐1, and GAPDH (see Table 2). The 2^ΔΔ^Ct method was used to calculate the relative gene expression quantity, normalized to GAPDH, which served as an internal control.

### 5.16 Immunofluorescence Staining (IF)

PC12 cells were seeded on coverslips and cultured in 24‐well plates for cellular IF. After pretreatment, the cells were fixed in 4% paraformaldehyde for 30 min and then permeabilized with 0.5% Triton X‐100. Spinal cord tissue biopsies were dewaxed, embedded in paraffin, hydrated and subjected to antigen retrieval using ethylenediamine tetraacetic acid buffer (pH 8.0). The tissue biopsies were blocked with 10% bovine serum albumin to prevent nonspecific antibody binding. The cells and tissue biopsy samples were incubated at 4°C with the following antibodies: anti‐γ‐glutamyltransferase 1 (GGT1) (1:1000, Affinity, Cat# DF6610, RRID: AB_2838572), anti‐microtubule‐binding protein 2 (MAP2) (1:500, Proteintech, Cat# 17490‐1‐AP, RRID: AB_2137880), neurofilament protein‐200 (NF200) (1:5000, Proteintech, Cat# 17490‐1‐AP, RRID: AB_2137880), anti‐neurofilament protein‐200 (NF200, 1:5000, Proteintech, Cat# 60331‐1‐IG, RRID: AB_2881440), anti‐stathmin2 (STMN2) (1:2000, Proteintech, Cat#67204‐1‐IG, RRID:AB_2882497), anti‐F4/80 (1:1000, Affinity, Cat#DF6332, RRID:AB_2838296), anti‐oligodendrocyte transcription factor 2 (Olig2) (1:1000, Proteintech Cat# 13999‐1 – AP, RRID: AB_2157541), anti‐NeuN (1:2000, Proteintech Cat# 26975‐1 – AP, RRID: AB_2880708), anti‐coiled‐coil, moesin‐like BCL‐interacting protein (Beclin‐1) (1:1000, Proteintech, Cat#1306‐1‐AP, RRID:AB_2259061), anti‐p62/Sequestosome‐1 (SQSTM/p62) (1:500, Affinity, Cat#AF5384, RRID:AB_2837869), and anti‐microtubule‐associated protein 1 light chain 3 (LC3) (1:1000, Proteintech, Cat#14600‐1‐ AP, RRID:AB_2137737). The following day, the samples were incubated at room temperature for 30 to 60 min with the following secondary antibodies: Coralite488‐conjugated goat anti‐mouse IgG (H+L) (1:400, Proteintech, Cat# SA00013‐1, RRID: AB_2810983) and coralite594‐conjugated goat anti‐rabbit IgG (H+L) (1:400, Proteintech, Cat# SA00013‐4, RRID: AB_2810984). Then, nuclei were stained with 4', 6‐2 amino‐2‐phenyl indole (Servicebio, through Guangdong, China; Cat # G1012). The IF images were captured using a scanner (Pannoramic DESK P‐MIDI P250; 3 dHISTECH; Budapest, Hungary). The fluorescence sginal was quantified by measuring the intensity and percentage of fluorescence in three distinct regions of interest.

### 5.17 Histological Analysis

The spinal cord was dissected from 0.5 cm above to 0.5 cm below the site of SCI and subsequently subjected to histological analysis. Hematoxylin and eosin staining kits (Cat# G1120, SoraBio, Beijing, China) and a Nissl staining kit (Cat# G1434, SoraBio, Beijing, China) were obtained from SoraBio (Beijing, China). HE staining and Nissl staining were performed in strict accordance with the manufacturer's guidelines. After staining, each biopsy sample was dehydrated in ethanol and cleared with xylene for 10 min, and sealed with a neutral resin. The stained sections were visualized using a scanning device (Pannoramic DESK P‐MIDI P250).

### 5.18 Measurement of GSH/GSSG

PC12 cells were seeded in 6‐well plates. After the cells adhered, those in the control group were left untreated, while those in the OGD group were treated with OGD for 4 h, as previously described. Additionally, cells in the Enocyanin+OGD group was pretreated with 0.5 mg/mL Enocyanin for 2 h before being subjected to OGD for 4 h. GSH/GSSG levels were measured after 6 h, strictly following the instructions provided by the manufacturers of the GSH and GSSG assay kits.

### 5.19 Flow Cytometry

An Annexin V‐AbFluor 488/PI double‐staining apoptosis detection kit (Abbkine, China) was used for flow cytometry analysis to determine the rate of cell apoptosis. Data were acquired and analyzed using a BECKMAN COULTER CytoFLEX.

### 5.20 Reactive Oxygen Species (ROS) Detection

ROS generation was assessed using a ROS detection kit (Beyotime, Shanghai, China) according to the manufacturer's instructions. The ROS probe was dissolved in DMEM at a concentration of 10 µM and added to the cells. After 30 min of inculcation at 37°C, the cells were washed three times with DMEM. IF images were then captured using a scanner (Pannoramic DESK P‐MIDI P250; 3 dHISTECH; Budapest, Hungary). Fluorescence signals were quantified by measuring the intensity and percentage of fluorescence in three distinct regions of interest.

### 5.21 Mitochondrial Membrane Potential (*Δψm*) Measurement

The researcher utilized a JC‐1 staining detection kit from Beyotime (Shanghai, China). Following the kit's instructions, PC12 cells were incubated with the JC‐1 reagent for 20 min, washed, and then treated with the JC‐1 buffer. A fluorescence microscope produced by Zeiss (German) was employed to observe the cells. When the *Δψm* is high, JC‐1 accumulates in the mitochondrial matrix to form polymers, emitting red fluorescence (λex = 525 nm; λem = 590 nm); when the *Δψm* is low, JC‐1 remains as a monomer, emitting green fluorescence (λex = 490 nm; λem = 530 nm).

### 5.22 Surface Plasmon Resonance (SPR) Analysis

The surface plasmon resonance analysis was performed using the Biacore T200 instrument (Cytiva, Marlborough, MA, USA). Single‐cycle kinetics were employed to assess the binding behavior. Sensorgram baselines were normalized to zero and aligned with the injection start time, and reference flow cell signals were subtracted to account for non‐specific binding. The binding kinetics were evaluated using a 1:1 interaction model, yielding the association rate constant (ka) and dissociation rate constant (kd). Data analysis was conducted using the Biacore software (Cytiva).

### 5.23 In vitro BSCB Permeability Studies

bEnd.3 cells (CL‐0598, Wuhan Ponsaic Biotechnology Co., Ltd., China) were seeded at a density of 1 x 10^5^ cells per insert in the upper chamber of a Transwell apparatus and cultured to form a monolayer. The integrity of the monolayer was confirmed by achieving a transendothelial electrical resistance (TEER) of over 200 Ω/cm2. DiO‐labelled nanoparticles were applied to the upper chamber and incubated for 12 h. The permeability of the nanoparticles across the BSCB was quantified by measuring the fluorescence in the lower chamber using a spectrophotometer.

### 5.24 Transmission Electron Microscopy (TEM)

PC12 cells were fixed overnight with 10% glutaraldehyde, followed by a 2‐h fixation with 1% osmic acid. After washing with ethanol and acetone, the cells were rehydrated through a series of PBS solutions. The samples were then embedded in ethoxy resin, and sliced into in ultrafine sections, double‐stained with uranyl acetate and lead citrate, and observed under a transmission electron microscope (Hitachi, H‐7650).

### 5.25 Co‐Immunoprecipitation (IP) Assay

The 293T cells was used for the CO‐IP assay. Cells were lysed using a lysate buffer containing a cocktail of 1 mm PMSF,1 mM dithiothreitol (DTT), and a protease inhibitor. Cell lysates were immunoprecipitated with an anti‐Flag (1:100, Proteintech, Cat#66008‐4‐IG, RRID:AB_2918475), at 4°C overnight, followed by incubation with protein A/G‐beads for 4 h at 4°C. Subsequently, the complex was cleaned 5 times with PBS containing the protease inhibitor at 4°C. Bound proteins were eluted with 10% SDS buffer and analyzed by WB.

### 5.26 CETSA

Cells were treated with either DMSO or enocyanin for 2 h. Subsequently, they were washed three times with PBS to remove any residual substances. The cells were then divided into equal parts and subjected to heat treatment at different temperatures for 3 min. After that, a freeze—thaw process was conducted three times to gently break open the cells. Finally, western blotting was used to analyze the cellular contents, aiming to observe any changes in the cells after the treatments.

### 5.27 Surface Plasmon Resonance (SPR) Analysis

Surface plasmon resonance (SPR) detection was performed using the Biacore T200 instrument (Cytiva, Marlborough, MA, USA). Single‐cycle kinetics were employed to assess the binding behavior. Sensorgram baselines were normalized to zero and aligned with the injection start time, and reference flow cell signals were subtracted to account for non‐specific binding. The binding kinetics were evaluated using a 1:1 interaction model, yielding the association rate constant (ka) and dissociation rate constant (kd). Data analysis was conducted using the Biacore software (Cytiva).

### 5.28 Statistical Analysis

Statistical comparisons of two independent groups were performed using unpaired Student’ t‐test. The one‐way analysis of variance (ANOVA) was used with Bonferroni post hoc correction for multiple comparisons. All the data were analyzed by GraphPad Prism v.9.0. All research data were obtained from a minimum of three independent experiments. Statistical significance was determined at a threshold of *p* < 0.05. Error bars represent the standard deviation for parametric data and the calculated 95% confidence intervals for nonparametric data.

## Author Contributions

T.Yang, L.Y.,P.X, and T,S. contributed equally to this work. T.You, S.T. and F.Z. were involved in the conception, design, and supervision of the study. T.Yang, L.Y., P.X., and T.S. were involved in the development of methodologies, acquisition, analysis, and interpretation of data. J.C., H.Z., Z.L., X.C., and J.D. were involved in the performance of experiments, data collection, and analysis. X.D., A.S., B.D., and M.W. were involved in the reagents, materials, and analysis tools contribution. T.Yang finished the manuscript writing. T.You, S.T., and F.Z. edited the paper.

## Funding Sources

This work was supported by the Key Scientific Research Project of Anhui Provincial Health Commission (grant number AHWJ2024Aa10176); and the Natural Science Research Project of Anhui Province University (grant number 2023AH040394); the Hefei Comprehensive National Science Center Leading Medicine and Frontier Technology Research Institute Project (grant number 2023IHM01073); the National Natural Science Foundation of China (grant number 82502859); the Shanghai Natural Science Foundation (grant number 25ZR1402424); the Science and Technology Plan Project of Taizhou (grant numbers 25ywa06).

## Conflicts of Interest

The authors declare no conflicts of interest.

## Supporting information




**Supporting File 1**: advs75554‐sup‐0001‐SuppMat.docx.

**Supporting File 2**: advs75554‐sup‐0002‐FigureS1‐S8.zip.

## Data Availability

The data used to support the findings of this study are available from the corresponding author upon request.
