# Supplementary material for: “Membrane‐Guided” Repair Strategy: Precision Delivery of GGT1 Degrader for Targeted Repair and Regeneration of Spinal Cord Neurons
Source: Adv Sci (Weinh). 2026 May 14;13(42):e75554. doi: 10.1002/advs.75554 (PMC13336134; doi:10.1002/advs.75554)
Supplement: Supplementary file 1 — Supporting File 1: advs75554‐sup‐0001‐SuppMat.docx. [file ADVS-13-e75554-s002.docx]

**Supplementary information**

Figure. S1 Detailed of drug library screening.

Figure.S2 Structure, cytotoxicity assay, and gene regulation detection of EA.

Figure. S3 EA ameliorates neuronal damage and restores motor function in SCI model mice.

Figure. S4 Transcriptomic Profiling of EA-Treated PC12 cells and the corresponding enrichment analysis results.

Figure. S5 Statistical charts corresponding to some results in Fig. 3.

Figure. S6 Screening for ubiquitin-related proteins that interact with GGT1.

Figure. S7 Evaluation of NSCm@EA treament efficacy *in vitro.*

Figure. S8 Deciphering neuronal heterogeneity and modulation: Insights from differentially expressed genes and pathway activity.
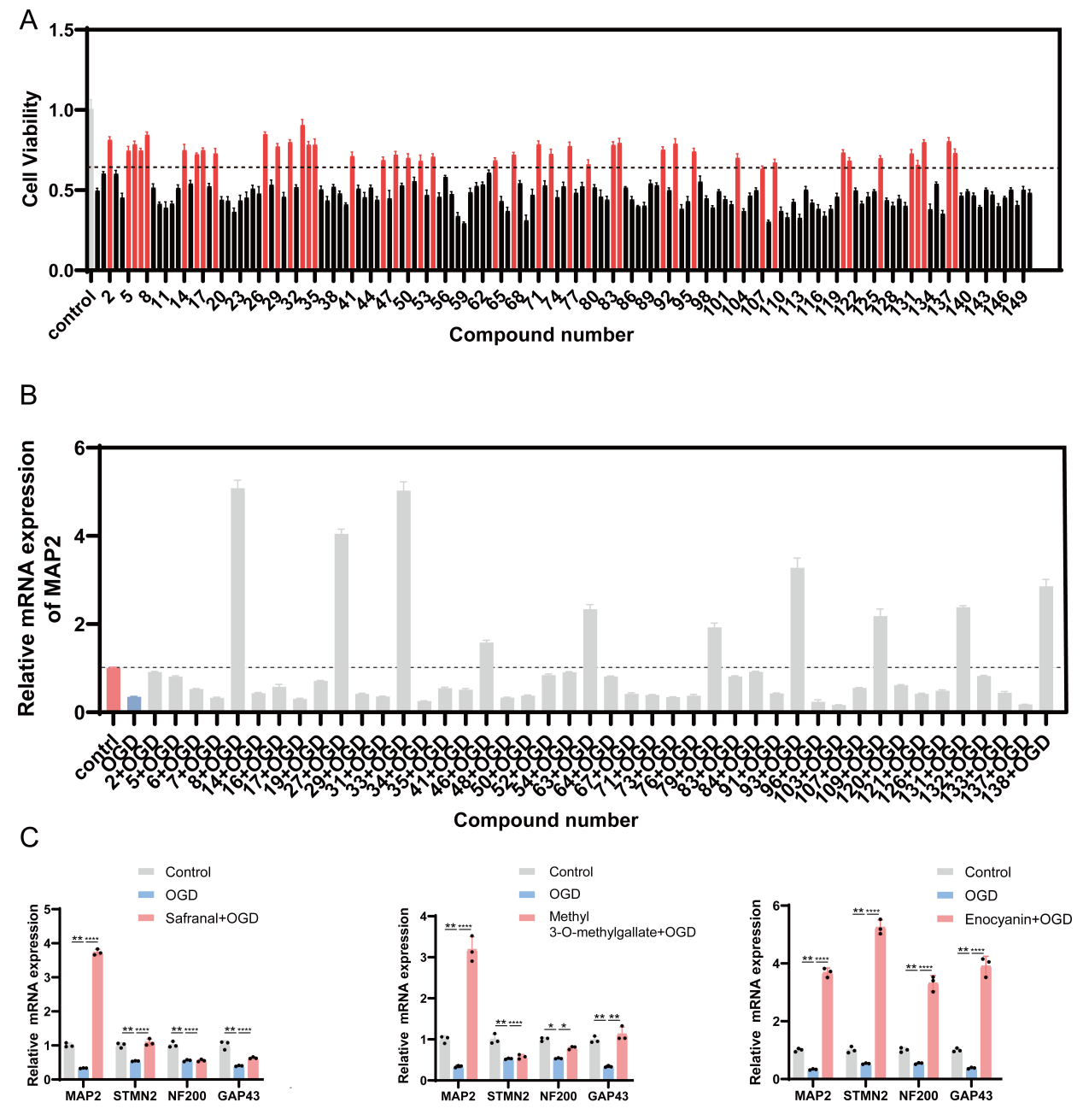


Figure. S1 Detailed of drug library screening. (A) 2mM drug for 24h after Erastin stimulation of neural stem cells, cell activity with CCK8 measurement. (B) Neural stem cells induced by OGD were treated with 2mM drug for 24h, and the expression of MAP2 was detected by RT-qPCR. (C) RT-qPCR demonstrated the regulatory effects of three candidate drugs on MAP2, STMN2, NF200, and GAP43 mRNA under OGD culture conditions. Data are expressed as mean ± SD, All are comparisons between each group and control, *: P < 0.05, **: P < 0.01 and ***: P < 0.001.


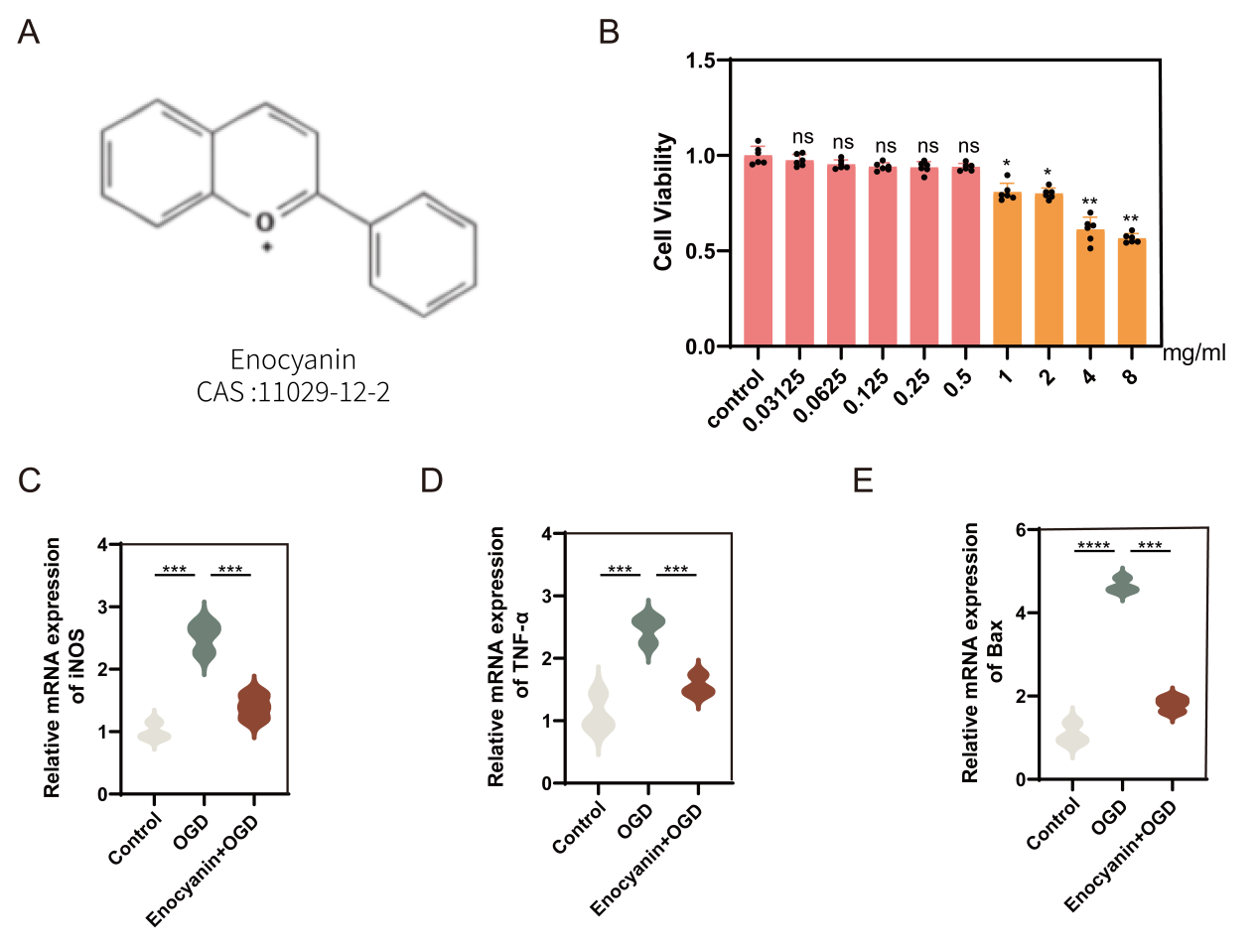
Figure. S2 Structure, cytotoxicity assay, and gene regulation detection of EA.

(A) Chemical structure of Enocyanin. (B) Enocyanin drug concentration of screening. (C-E) The expression levels of iNOS, TNF-α and Bax in PC12 cells treated with enocyanin were detected by RT-PCR. Data are expressed as mean ± SD, All are comparisons between each group and control, *: P < 0.05, **: P < 0.01 and ***: P < 0.001.


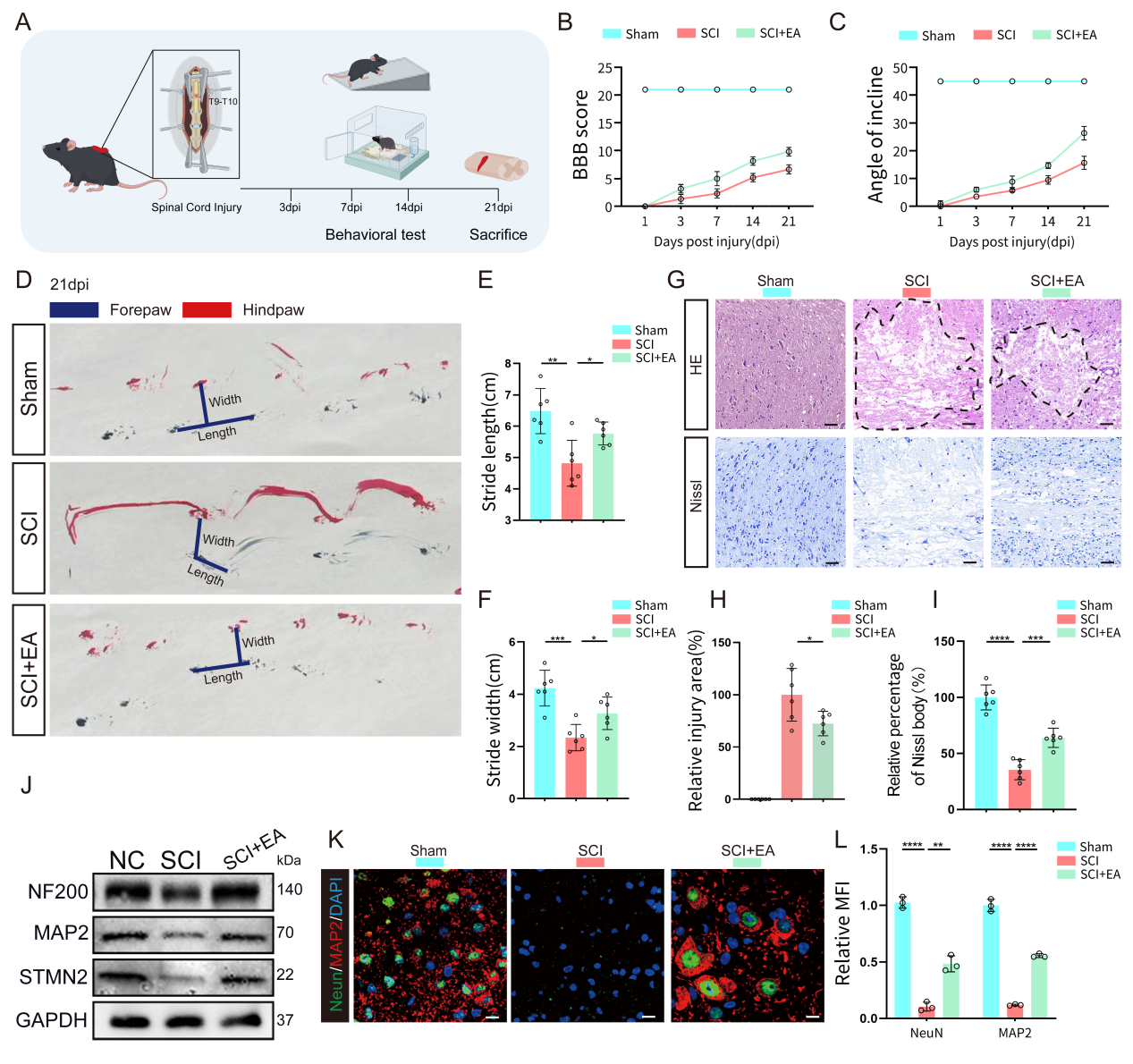


Figure. S3 EA ameliorates neuronal damage and restores motor function in SCI model mice. (A) Schematic of in vivo experimental design. (B-C) BBB scores and oblique plate Angle at 1,3,7,14 and 21 days after SCI (n=6). (E-F) Statistical Analysis of Step Length and Step Width. (G) HE staining and Nissl staining of spinal cord longitudinal sections of mice 21 days after sci. Scale bars: 50μm.(H, I) Statistical analysis of HE and Nissl staining (n=6). (J) WB detected of spinal cord tissue regeneration of axons marker protein expression level. (K-J) IF was used to detect the immunoreactivity of Neun (green-CoraLite488) and MAP2 (red-CoraLite594). (n=3).Scale bars:20μm. Data are expressed as mean ± SD, *: P < 0.05, **: P < 0.01 and ***: P < 0.001.


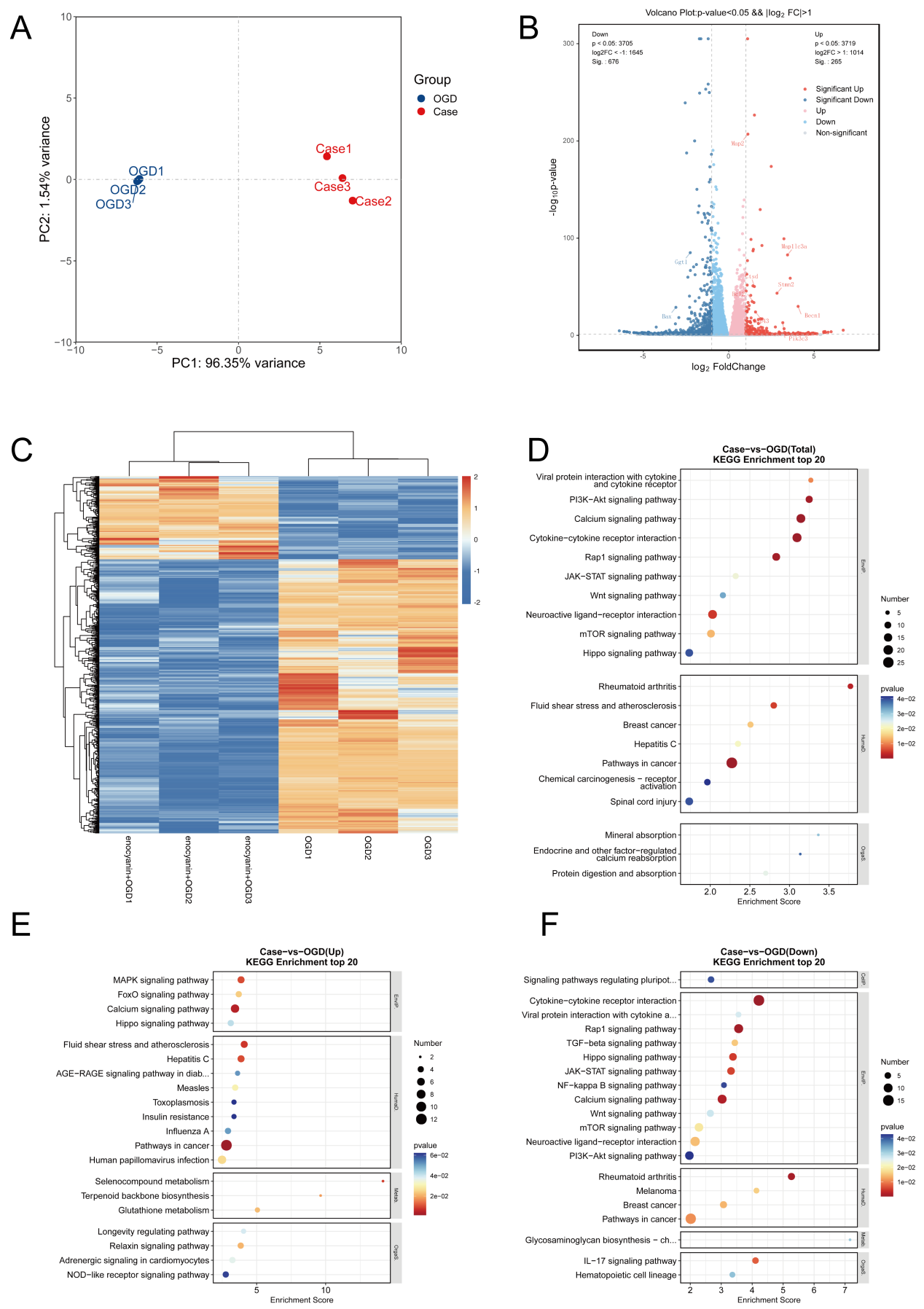


Figure. S4 Transcriptomic Profiling of EA-Treated PC12 cells and the corresponding enrichment analysis results. (A) The PCA plot illustrates the differences between the two groups in RNA-seq analysis, where the OGD group corresponds to the in vitro cell model, and the "Case" group corresponds to EA treatment after OGD modeling. (B) The volcano plot displays the differentially expressed genes (DEGs) between the two groups, with red representing up-regulated DEGs (Up-DEGs) and blue representing down-regulated DEGs (Down-DEGs). (C) The heat map shows all DEGs between the two groups, with a small subset of genes significantly down-regulated after EA treatment. (D) KEGG enrichment analysis was performed on all DEGs, with bubble plots displaying the top20 KEGG entries. (E) KEGG enrichment analysis was performed on all Up-DEGs, with bubble plots displaying the top20 KEGG entries. (F) KEGG enrichment analysis was performed on all Down-DEGs, with bubble plots displaying the top20 KEGG entries.


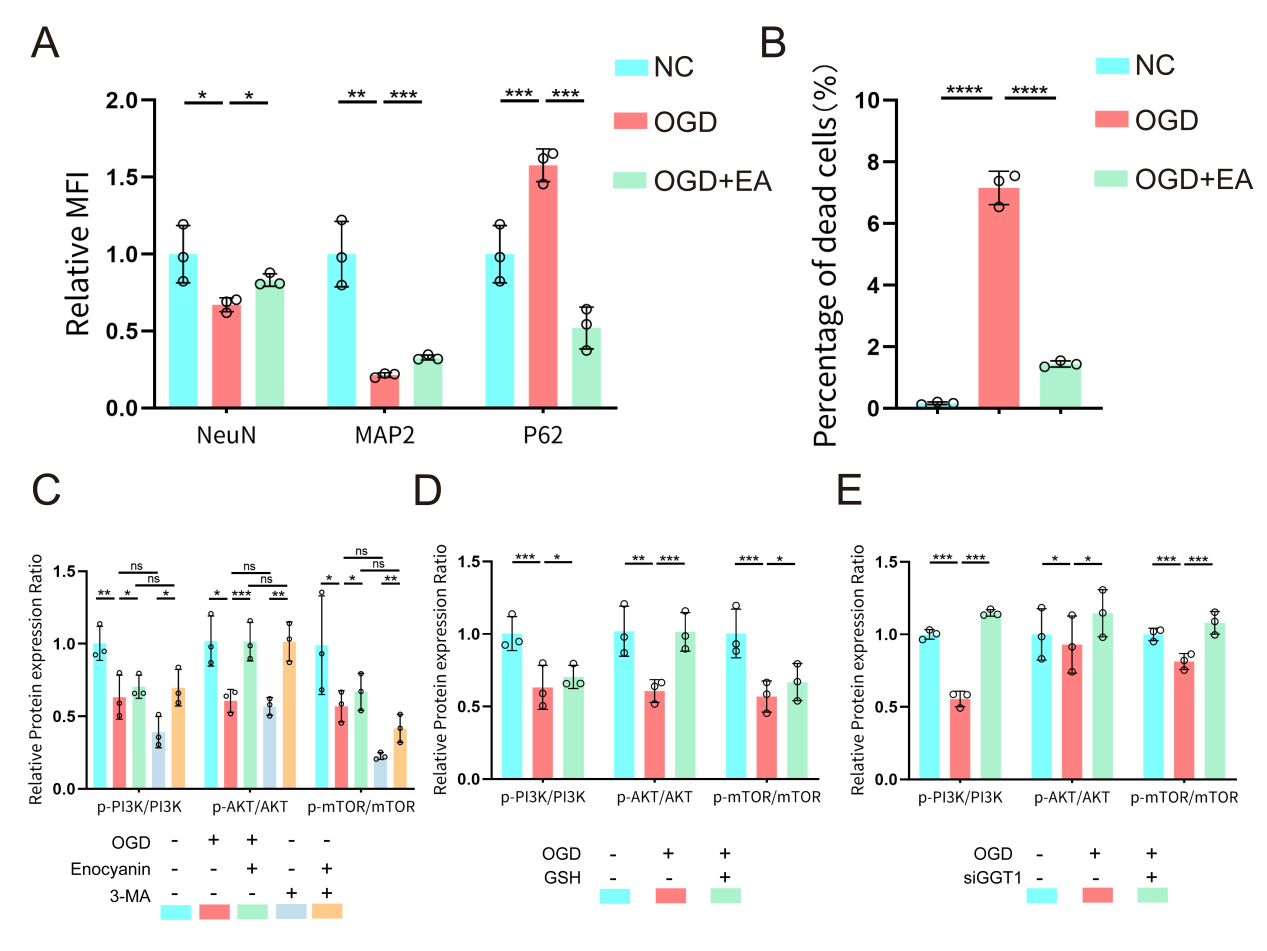
Figure. S5 Statistical charts corresponding to some results in Fig. 3. (A) Quantitative statistics were obtained regarding the expression of NeuN, COL2A1 and P62 (n=3). (B) Quantitative statistical flow cytometry for detecting the proportion of dead cells (n=3). (C-E) The activation of PI3K/AKT/mTOR signaling pathway was quantitatively analyzed after specific treatment (n=3). Data are expressed as mean ± SD, *: P < 0.05, **: P < 0.01 and ***: P < 0.001.

**
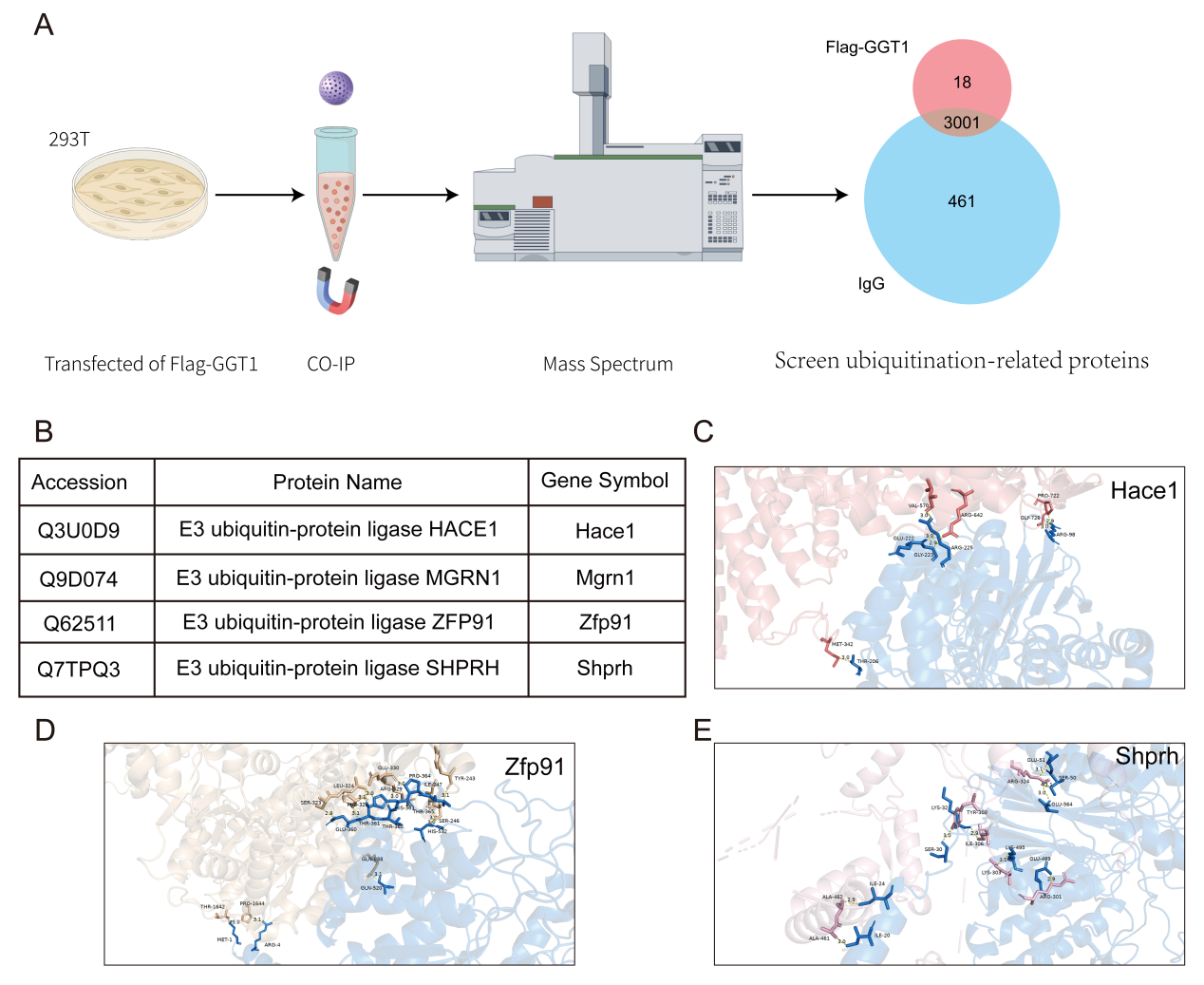
**Figure. S6 Screening for ubiquitin-related proteins that interact with GGT1. (A) Schematic diagram of IP-MS combined detection of GGT1 interacting proteins. (B) E3 ubiquitin ligase obtained by mass spectrometry. (C-E) Molecular docking of GGT1 with each E3 ubiquitin ligase.


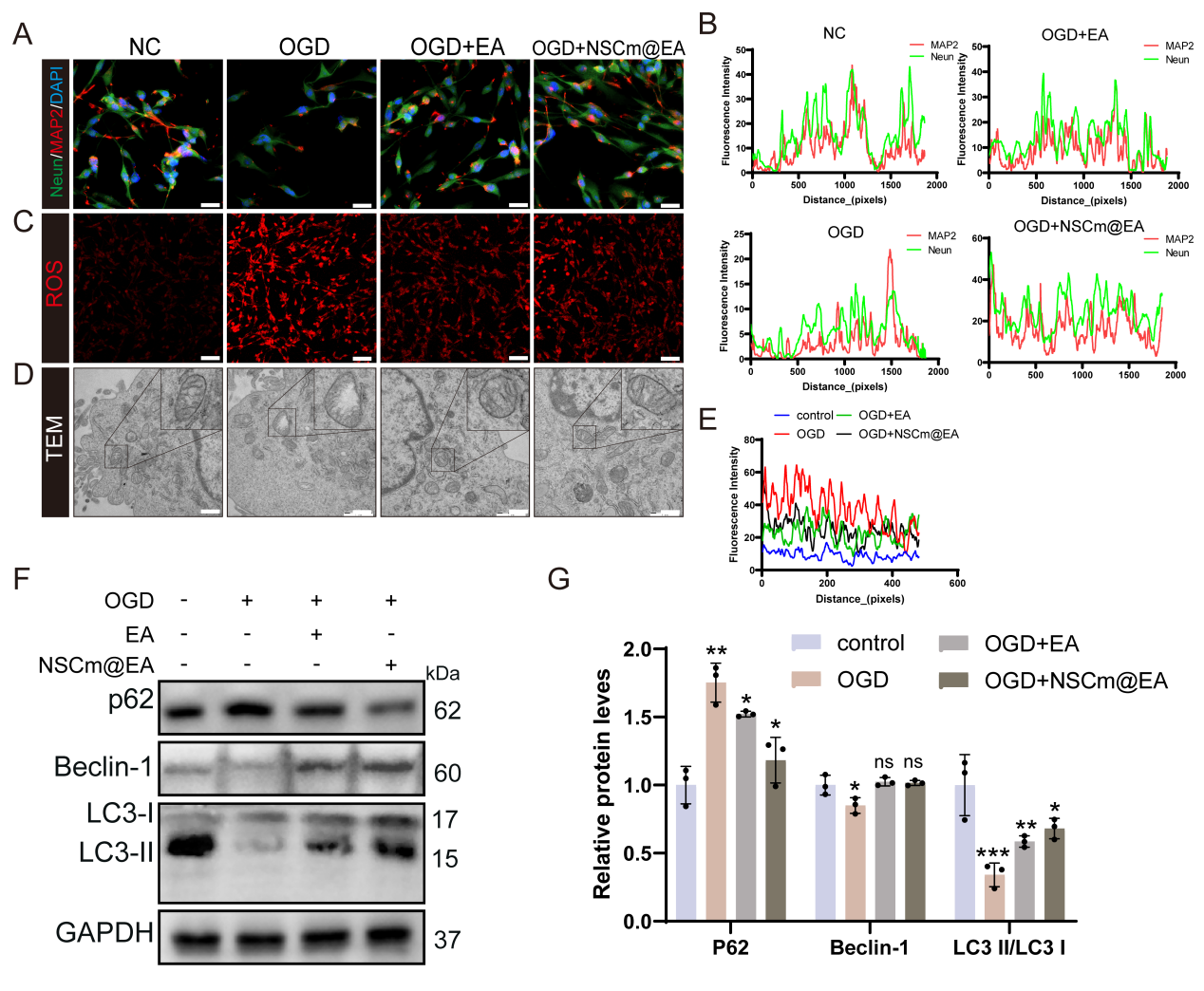
**Figure. S7** Evaluation of NSCm@EA treament efficacy *in vitro.* (A-B) IF detected Neun (green-CoraLite488), MAP2 (red-CoraLite594) in EA and NSCm@EA in PC12 cells. Scale bars: 20μm. (C) Determination of ROS, Scale bar: 100μm. (D) Mitochondrial morphology were measured by TEM, Scale bars:2μm or 1μm. (E) Quantitative analysis of fluorescence intensity of ROS. (F) WB detected autophagy marker p62, Beclin 1 and LC3-I/II in PC12 cells. (G) Quantitative analysis of ratio corresponding to protein bands. Data are expressed as mean ± SD, All are comparisons between each group and control, *: P < 0.05, **: P < 0.01 and ***: P < 0.001.


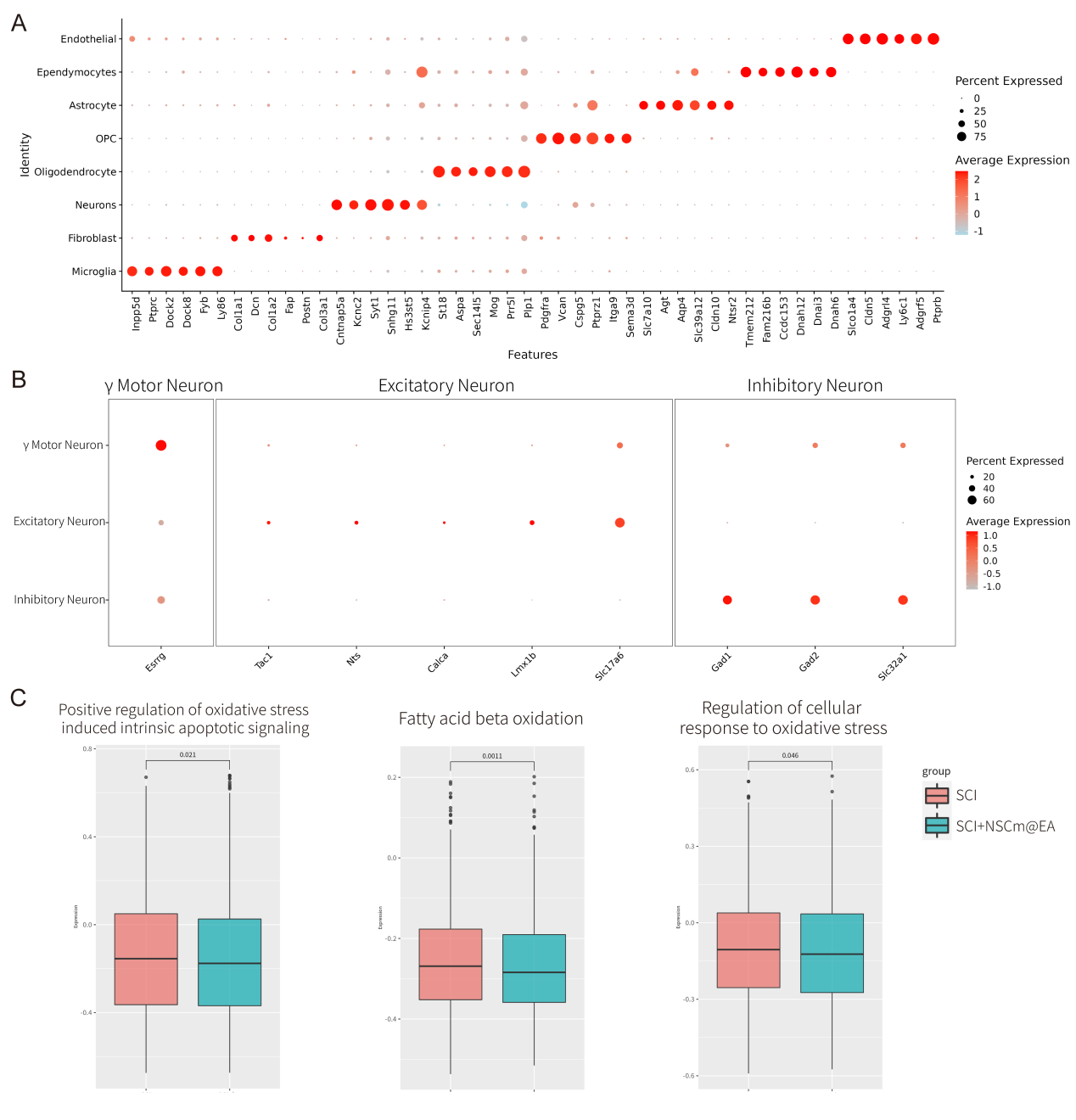
**Figure. S8** Deciphering neuronal heterogeneity and modulation: Insights from differentially expressed genes (DEGs) and pathway activity. (A) Dot plot of the top six DEGs for each cluster. The color of the dot indicates the average RNA expression of the gene in the cell type, and the size of the dot indicates the percentage of cells in the cluster that express the gene. (B) The cellmarker for two groups of neuronal subpopulation classifications showed by dot plot. (C) The box plot visually illustrates the negative regulatory effects of NSCm@EA treatment on neuronal cell apoptosis, fatty acid β-oxidation and oxidative stress pathways.


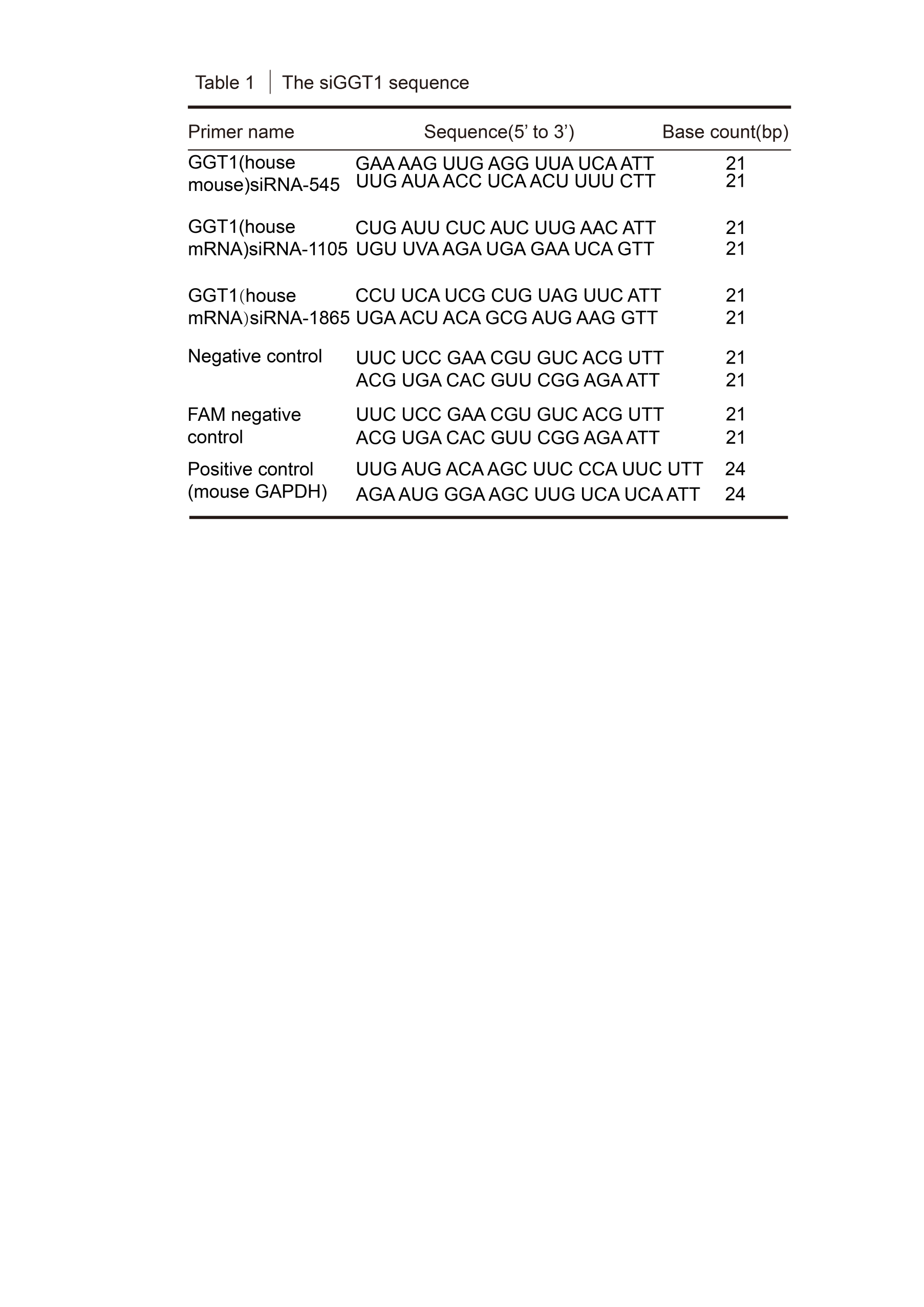
**Table 1** The SiGGT1 sequence used in this work.


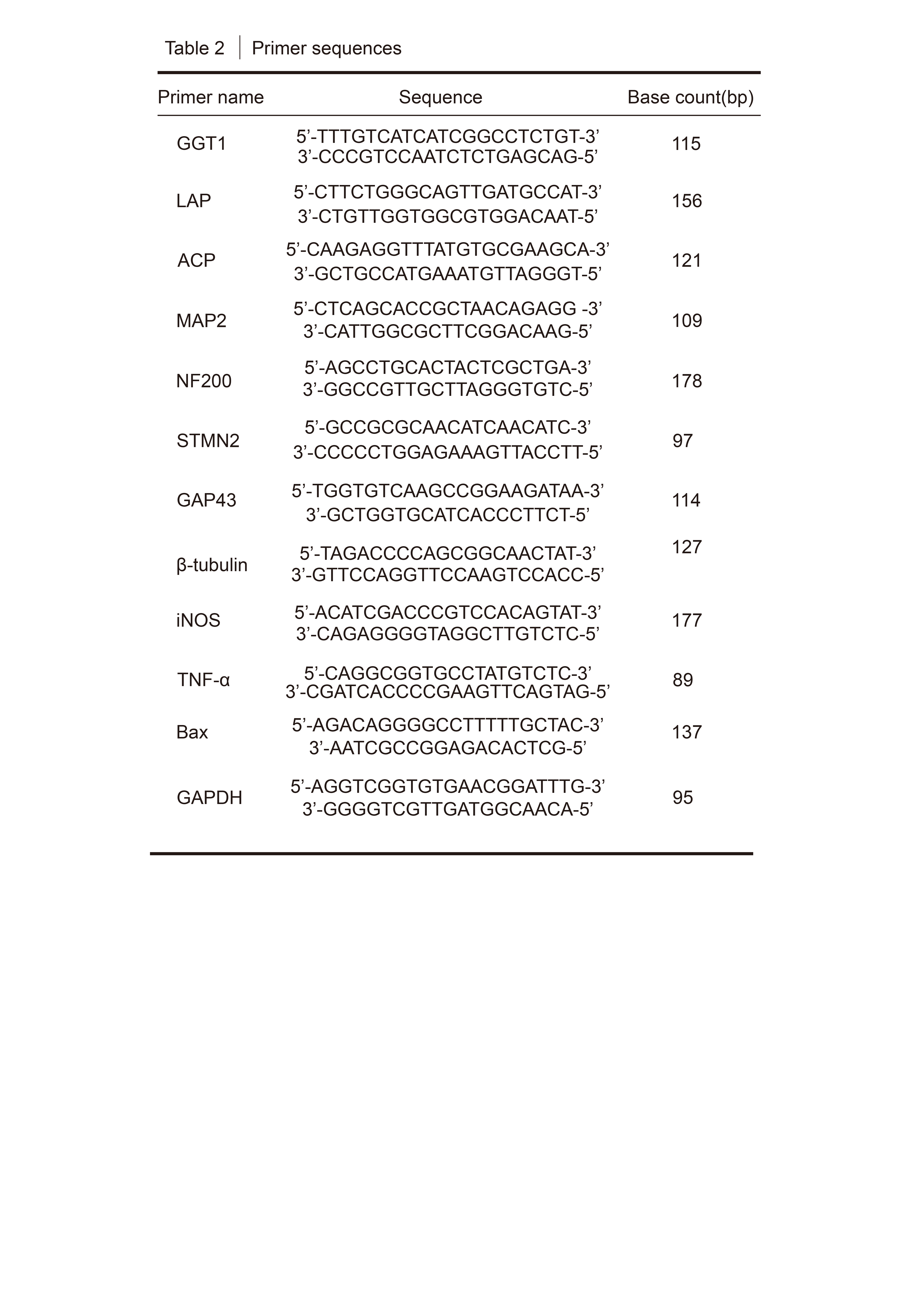


**Table 2** The gene primer sequence used for qPCR in this work
