# Supplementary figures and images for: “Membrane‐Guided” Repair Strategy: Precision Delivery of GGT1 Degrader for Targeted Repair and Regeneration of Spinal Cord Neurons
Source: Adv Sci (Weinh). 2026 May 14;13(42):e75554. doi: 10.1002/advs.75554 (PMC13336134; doi:10.1002/advs.75554)

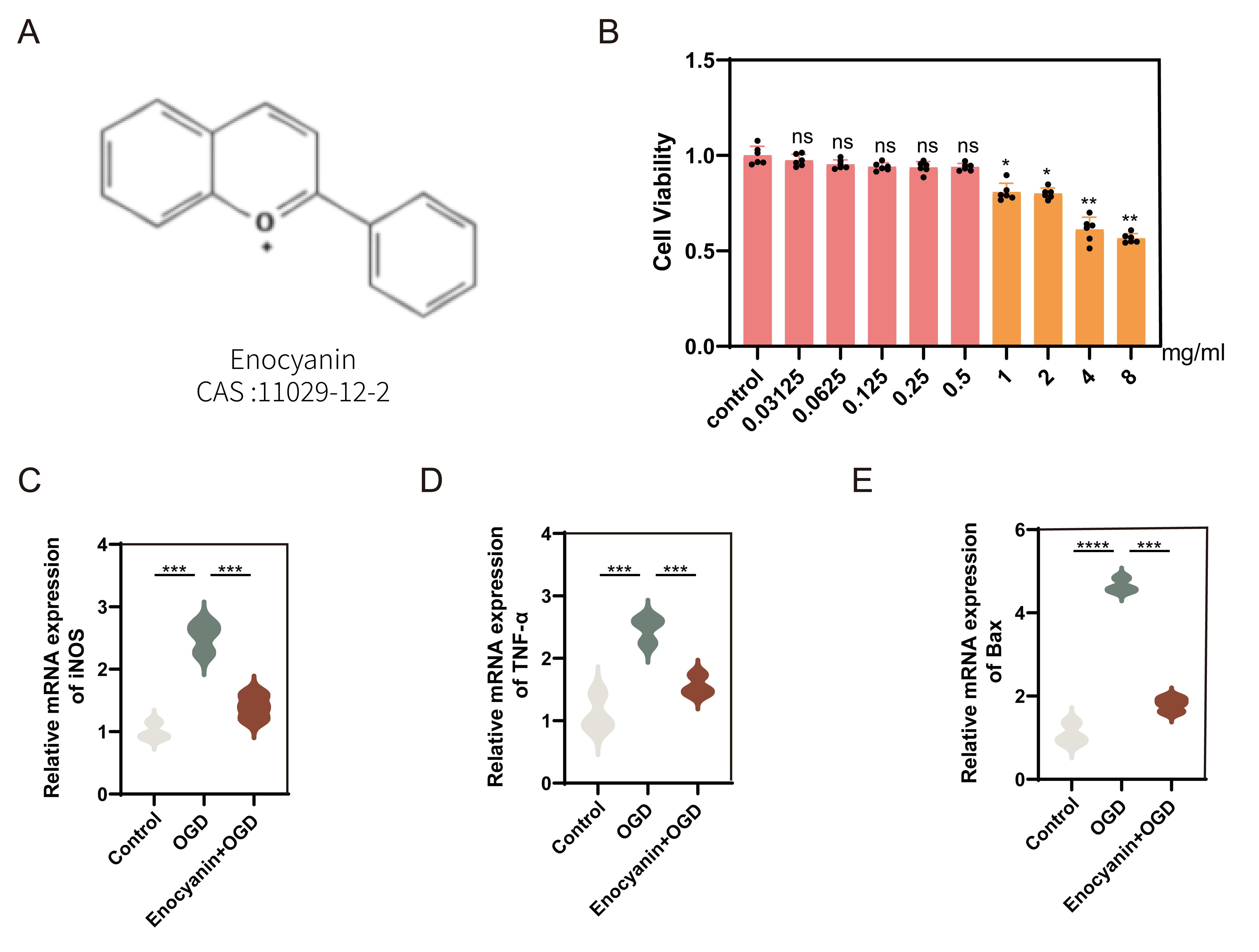

Supplement: Supplementary file 2 — Supporting File 2: advs75554‐sup‐0002‐FigureS1‐S8.zip. [file ADVS-13-e75554-s001.zip › sup-2-re.png]

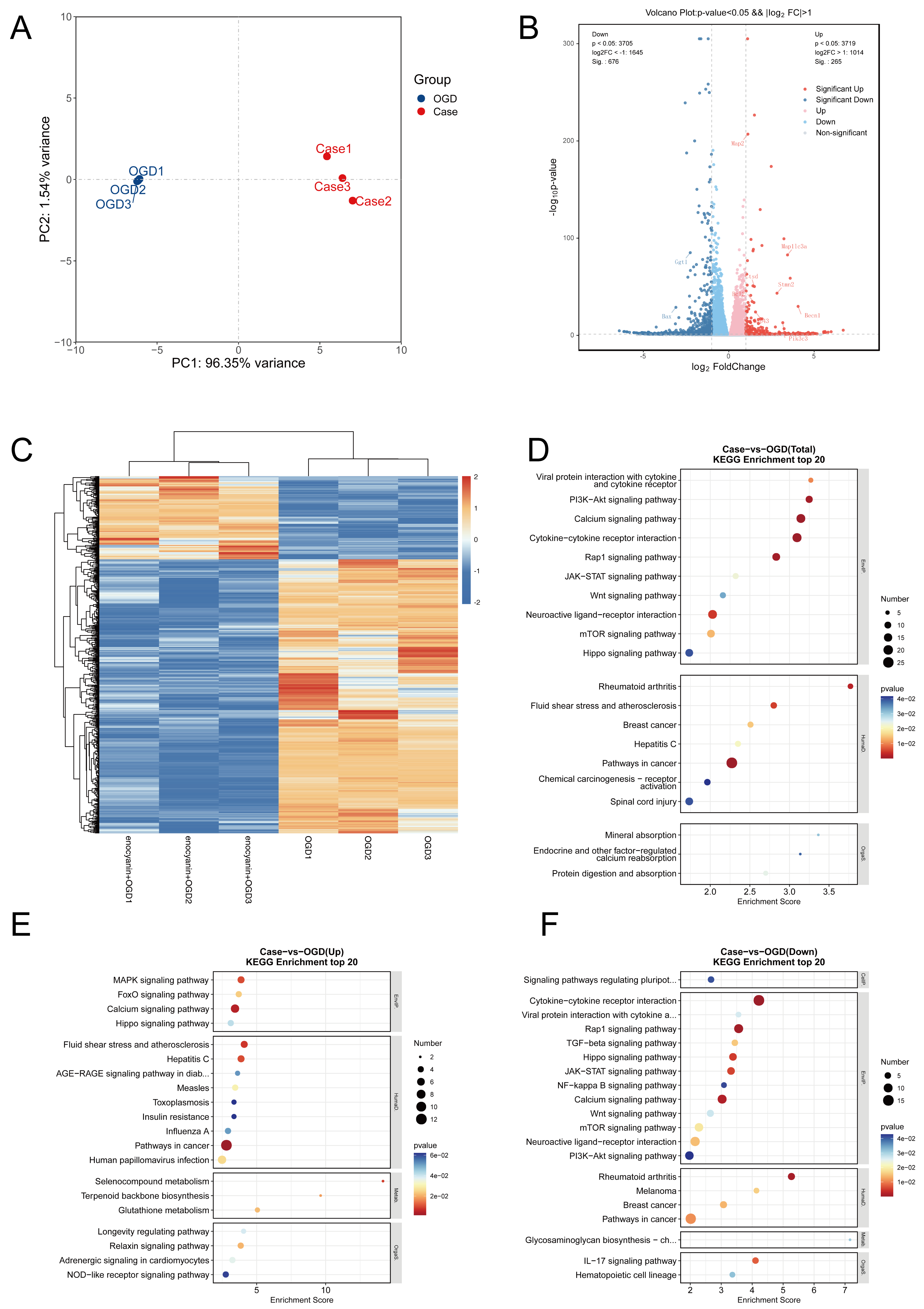

Supplement: Supplementary file 2 — Supporting File 2: advs75554‐sup‐0002‐FigureS1‐S8.zip. [file ADVS-13-e75554-s001.zip › sup-4.png]

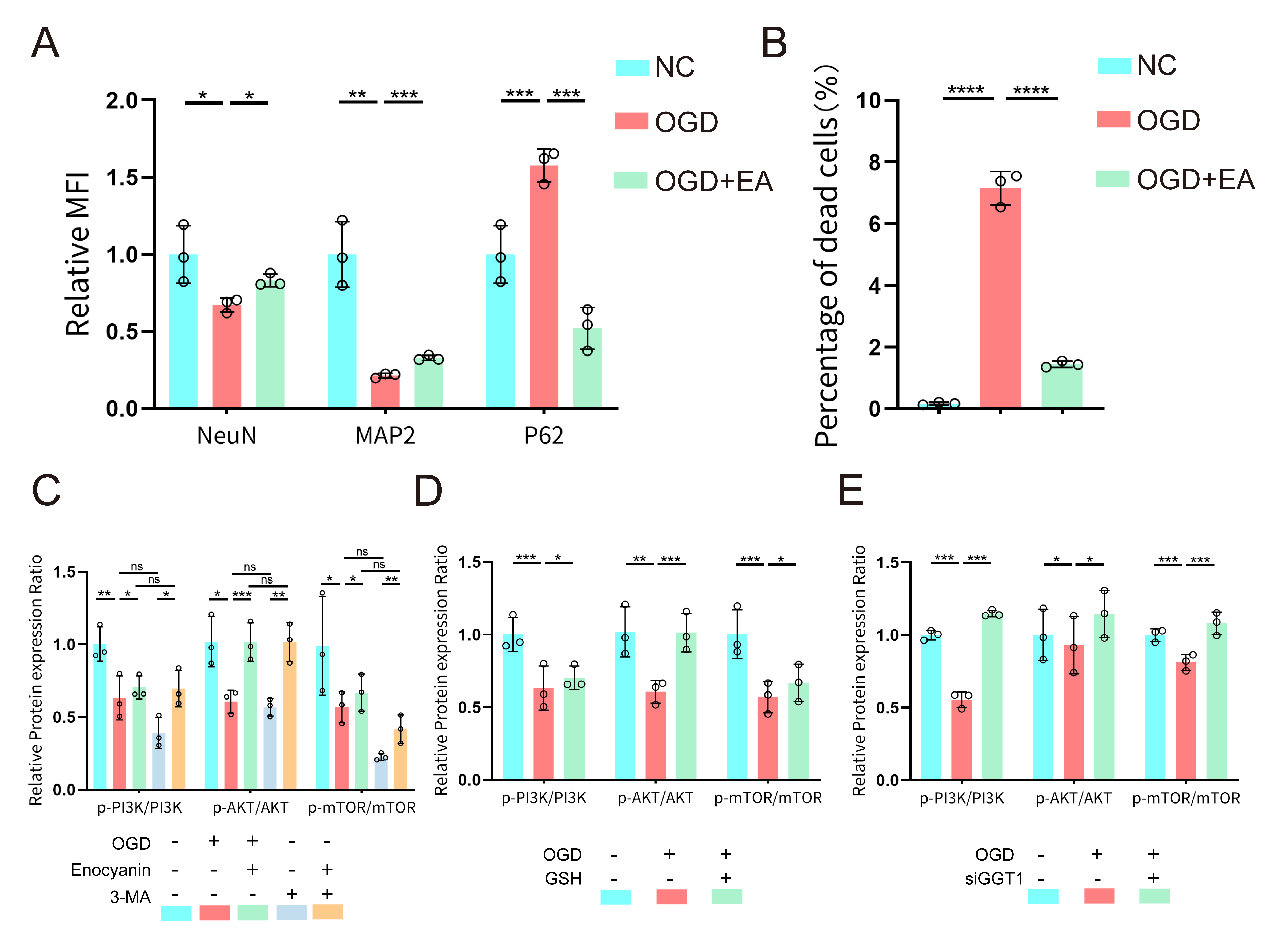

Supplement: Supplementary file 2 — Supporting File 2: advs75554‐sup‐0002‐FigureS1‐S8.zip. [file ADVS-13-e75554-s001.zip › sup-5-re.png]

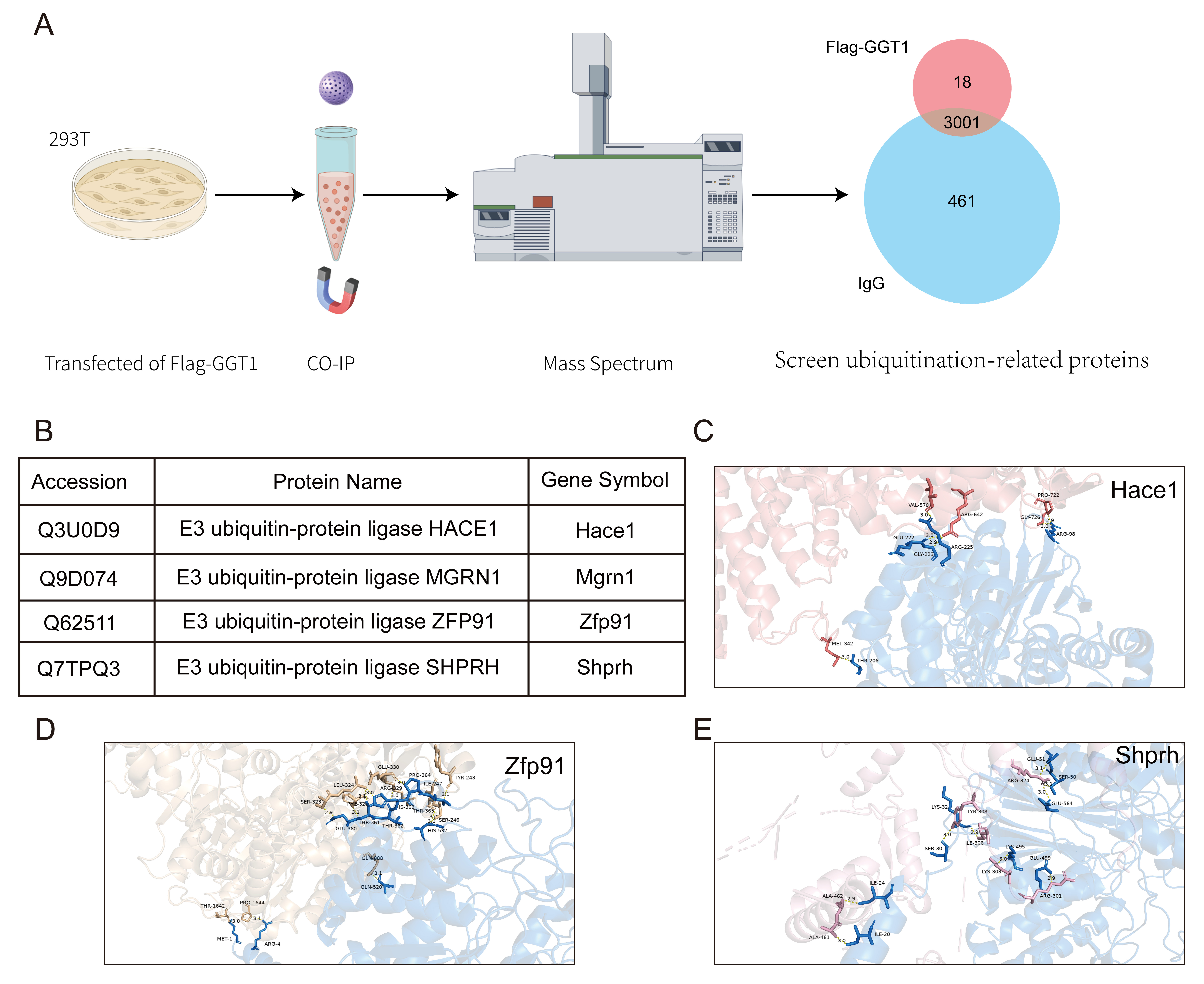

Supplement: Supplementary file 2 — Supporting File 2: advs75554‐sup‐0002‐FigureS1‐S8.zip. [file ADVS-13-e75554-s001.zip › sup-6.png]

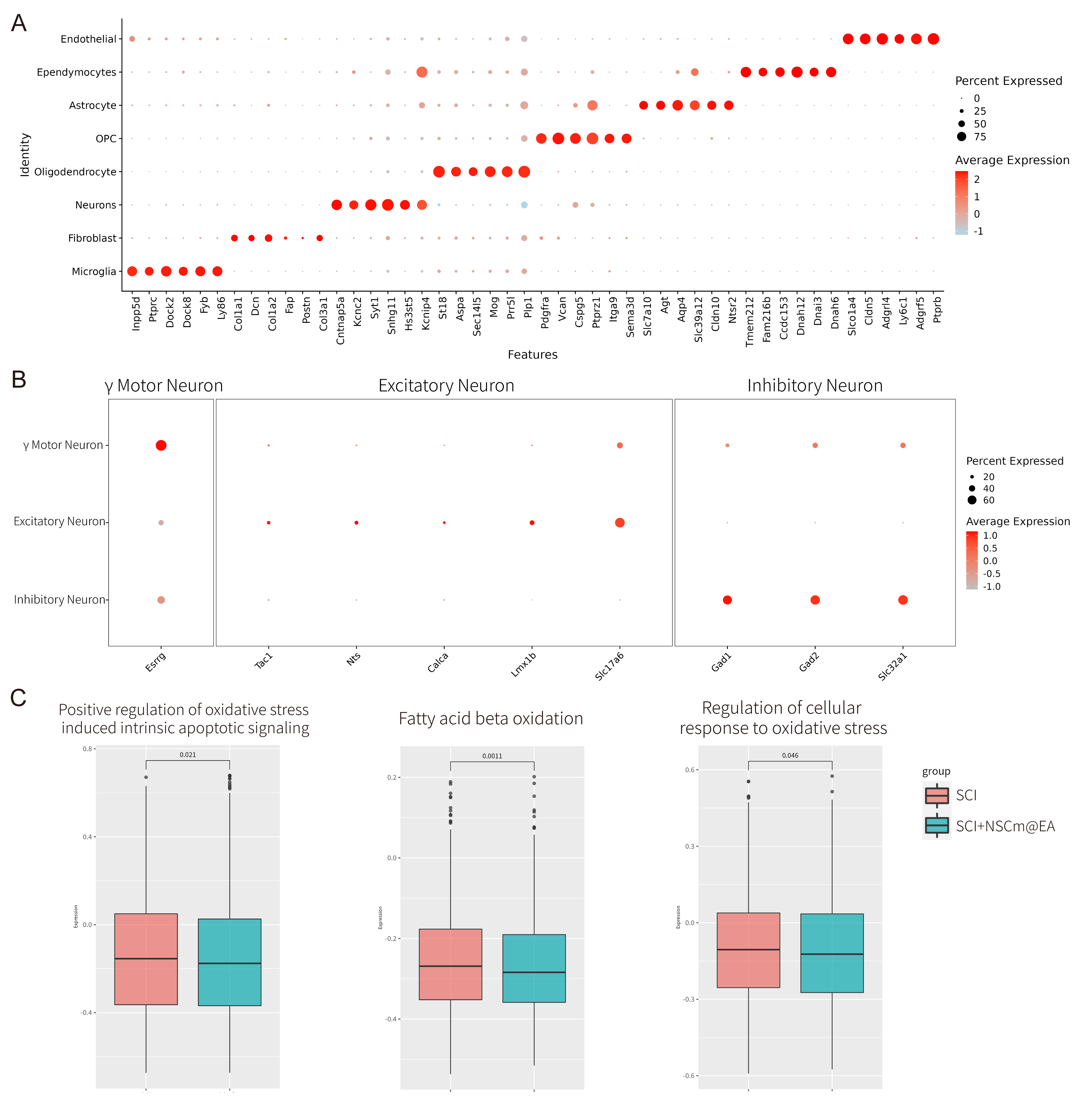

Supplement: Supplementary file 2 — Supporting File 2: advs75554‐sup‐0002‐FigureS1‐S8.zip. [file ADVS-13-e75554-s001.zip › sup-8.png]
